# Long noncoding RNA HITT coordinates with RGS2 to inhibit PD-L1 translation in T cell immunity

**DOI:** 10.1172/JCI162951

**Published:** 2023-06-01

**Authors:** Qingyu Lin, Tong Liu, Xingwen Wang, Guixue Hou, Zhiyuan Xiang, Wenxin Zhang, Shanliang Zheng, Dong Zhao, Qibin Leng, Xiaoshi Zhang, Minqiao Lu, Tianqi Guan, Hao Liu, Ying Hu

**Affiliations:** 1 School of Life Science and Technology, Harbin Institute of Technology, Harbin, China.; 2 Department of Breast Surgery, Harbin Medical University Cancer Hospital, Heilongjiang Academy of Medical Sciences, Harbin, China.; 3 BGI-SHENZHEN, Shenzhen, China.; 4 Affiliated Cancer Hospital and Institute of Guangzhou Medical University, State Key Laboratory of Respiratory Disease, Guangzhou, China.; 5 Department of Clinical Laboratory, Qilu Hospital of Shandong University, Jinan, Shandong, China.; 6 Key Laboratory of Science and Engineering for the Multi-modal Prevention and Control of Major Chronic Diseases, Ministry of Industry and Information Technology, Zhengzhou Research Institute of Harbin Institute of Technology, Zhengzhou, China.; 7 Department of Medicine and Health, Zhengzhou Research Institute of Harbin Institute of Technology, Zhengzhou, China.

**Keywords:** Immunology, Oncology, Cancer immunotherapy

## Abstract

Programmed cell death ligand 1 (PD-L1) is an immune checkpoint protein frequently expressed in human cancers that contributes to immune evasion through its binding to PD-1 on activated T cells. Unveiling the mechanisms underlying PD-L1 expression is essential for understanding the impact of the immunosuppressive microenvironment and is also crucial for the purpose of reboosting antitumor immunity. However, how PD-L1 is regulated, particularly at translational levels, remains largely unknown. Here, we discovered that a long noncoding RNA (lncRNA), HIF-1α inhibitor at translation level (HITT), was transactivated by E2F transcription factor 1 (E2F1) under IFN-γ stimulation. It coordinated with regulator of G protein signaling 2 (RGS2) in binding to the 5′ UTR of *PD-L1*, resulting in reduced PD-L1 translation. HITT expression enhanced T cell–mediated cytotoxicity both in vitro and in vivo in a PD-L1–dependent manner. The clinical correlation between HITT/PD-L1 and RGS2/PD-L1 expression was also detected in breast cancer tissues. Together, these findings demonstrate the role of HITT in antitumor T cell immunity, highlighting activation of HITT as a potential therapeutic strategy for enhancing cancer immunotherapy.

## Graphical abstract

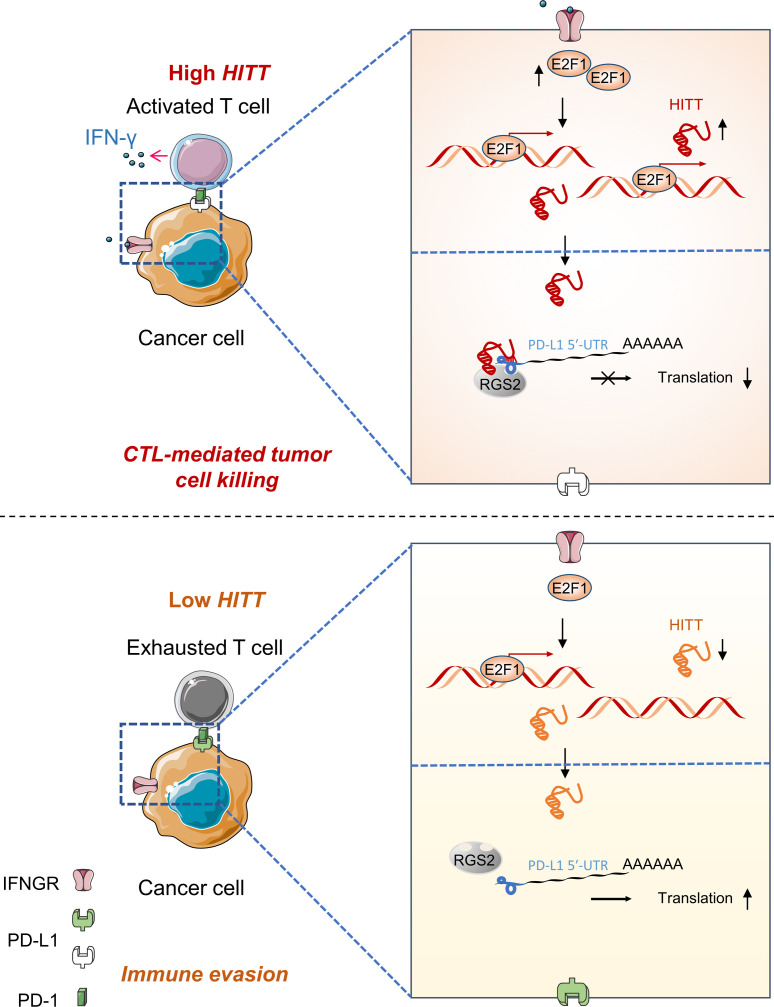

## Introduction

Immune escape is a hallmark of cancer evolution, involving a complex interplay between tumor cells and the host immune microenvironment, and is a central modifier of clinical outcomes (1). Cancer cells gain this fundamental trait by exploiting a plethora of immunosuppressive pathways, such as the induction of immune checkpoints, as exemplified by programmed cell death ligand 1 (PD-L1) (2). PD-L1 binds with programmed cell death 1 (PD-1), a key immune checkpoint protein expressed on the surface of activated T cells, leading to suppressed cytotoxic T cell activity (3). Unsurprisingly, immunotherapies that aim to achieve immune checkpoint blockade by targeting the PD-1/PD-L1 interaction have yielded striking clinical benefits in advanced malignancies (4, 5). Nevertheless, only a small fraction (20%–40%) of patients benefit from PD-1/PD-L1 blockade therapies (6). Compared with other genes, such as IFN-c, IDO1, and CXCL9, PD-L1 expression is considered as a relative reliable predictor of response to treatment (7), though with exceptions (8). Thus, it is essential that we understand how PD-L1 is regulated because it may lead not only to response predictors of PD-1/PD-L1 blockade, but also alternative strategies for targeting the PD-1/PD-L1 pathway. Recently, mounting evidence has suggested that PD-L1 expression is regulated at multiple levels; however, how translational processes influence PD-L1 protein output remains poorly understood (4).

Regulator of G protein signaling 2 (RGS2) belongs to a family of proteins that participate in the G protein cycle (9). Like its family members, RGS2 functions to inactivate G protein signaling by serving as a GTPase-activating protein (9, 10). This activity requires a canonical RGS domain that is shared by all family members (9, 10). In line with its role in inhibiting G protein signaling, RGS2-KO mouse studies have revealed that it is essential in the cardiovascular and central nervous systems (11, 12). However, G protein signaling cannot explain all of the physiological functions of RGS2, leading to extensive efforts to elucidate the molecular mechanisms of noncanonical RGS2 functions. Because of this, an increasing number of protein-binding partners, in addition to G protein, have been discovered (13). These additional functions, which include angiogenesis, migration, and chronic inflammation, have led to the discovery of RGS2’s role in cancer pathology (14, 15). Although the underlying mechanisms and pathological significance remain largely unexplored, a function of RGS2 in regulating mRNA translation has also been reported (16). Moreover, RGS2 has been shown to be induced in activated T cells and have a bronchoprotective role in a murine model of LPS-induced airway inflammation (17, 18). However, how RGS2 regulates T cell immunity and whether it has a role in the context of cancer immunity are not yet understood.

Long noncoding RNAs (lncRNAs) are a class of RNA arbitrarily defined as RNA molecules longer than 200 nucleotides with limited protein-coding potential (19). In-depth studies suggest that lncRNAs exert their biological activities by forming complexes with mRNA, DNA, or proteins (20). A growing body of work shows that lncRNAs are key regulators in diverse physiological and pathological contexts, including cancer (21). However, although much has been learned about the multiple functions of lncRNAs in cancer cell proliferation, apoptosis, invasion, and migration, little is known about their potential to regulate immune evasion (21).

Previous work by our group identified an lncRNA named HIF-1α inhibitor at translation level (HITT), also known as linc00637 or PPP1R13B divergent transcript (PPP1R13B-DT) (22). By analyzing The Cancer Genome Atlas (TCGA) database and in-house samples, we found HITT to be downregulated in multiple types of cancer and decreased HITT expression to be associated with advanced stages of colon, bladder, breast, and liver cancers. Mechanistically, HITT elicits remarkable antitumor effects by modulating cells’ responses to hypoxia and DNA damage through inhibiting HIF-1α synthesis and ATM activity, respectively (22, 23). It is also worth noting that, in addition to hypoxia and DNA damage, cancer cells are inevitably insulted under inflammatory microenvironment conditions. Proinflammatory cytokines, such as IFN-γ, TNF-α, granulocyte-macrophage colony-stimulating factor (GM-CSF), and IL-10 secreted in the inflammatory tumor microenvironment, are regarded as important triggers of PD-L1 expression (4, 24). This is in line with the well-established connection among inflammation, immune evasion, and carcinogenesis. Thus, it will be of interest whether and how HITT, as a cancer-related stress responder, is involved in regulating T cell immunity in cancer.

## Results

### HITT promotes T cell immunity.

**Figure 1:**
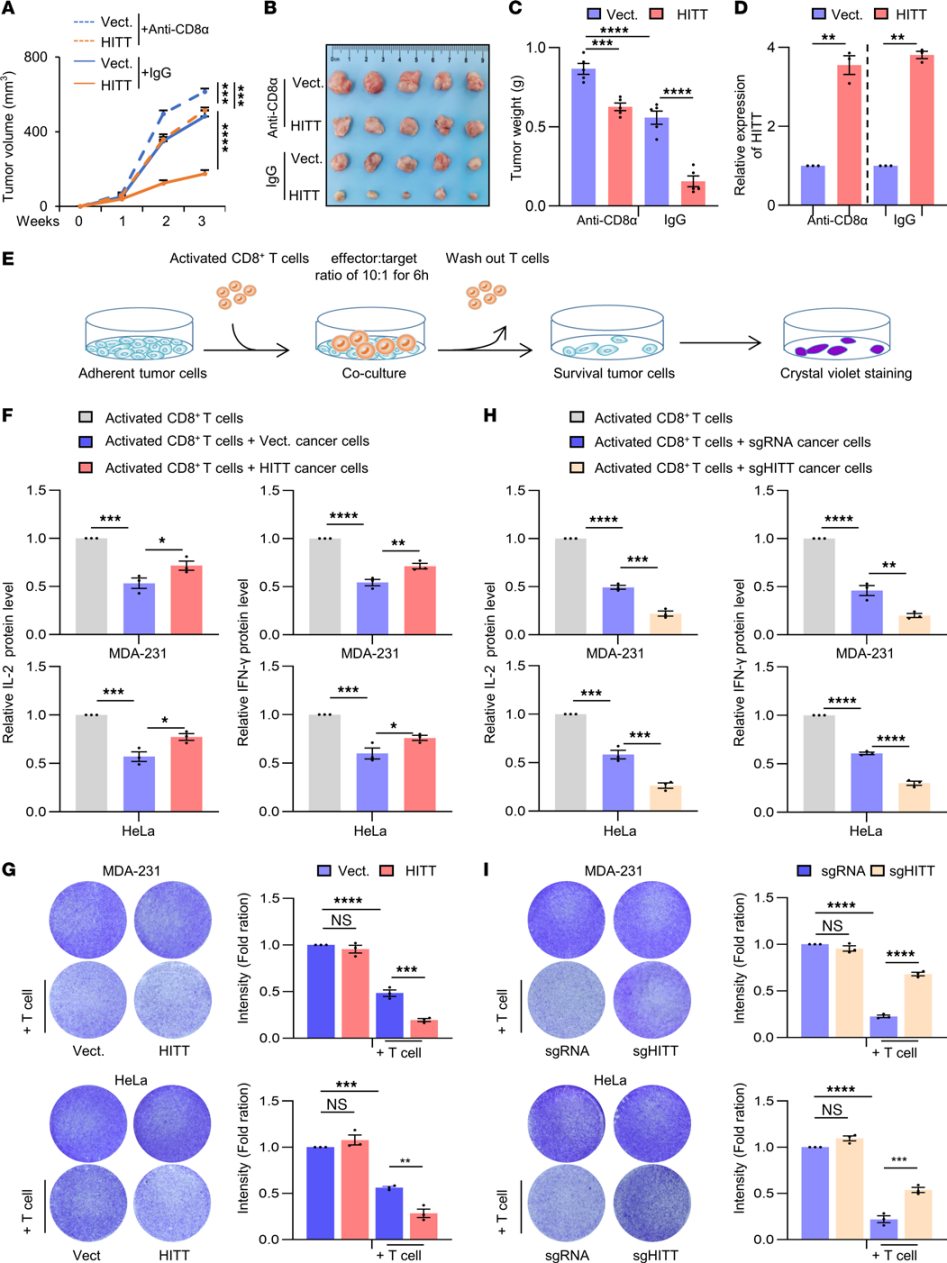
HITT sensitizes cancer cells to T cell–mediated cytotoxicity. (**A**–**C**) Volume (**A**), images (**B**), and weight (**C**) of 4T1 syngeneic tumors. Vect., vector. (**D**) HITT levels in 4T1 syngeneic tumors determined by qRT-PCR. (**E**) Schematic showing crystal violet staining to analyze T cell–mediated tumor cell–killing efficacy. (**F**) Detection of IL-2 and IFN-γ levels in the supernatants of T cell control and HITT-overexpressing MDA-231 and HeLa cell cocultures by ELISA assays. (**G**) Detection of the attached MDA-231 and HeLa cells by crystal violet staining after coculture with the activated T cells for 6 hours. Intensities are shown in bar graphs (right). (**H**) Detection of IL-2 and IFN-γ levels in the supernatants of T cell and MDA-231 and HeLa cell cocultures by ELISA assays. (**I**) Detection of the attached MDA-231 and HeLa cells by crystal violet staining after coculture with the activated T cells for 6 hours. Intensities are shown in bar graphs (right). Data in **A** and **C** are shown as mean ± SD (*n* = 5). Data in **C**, **D**, and **F**–**I** are derived from 3 independent experiments and are represented as mean ± SEM. *P < 0.05; **P < 0.01; ***P < 0.001; ***P < 0.0001; NS, not significant by 2-way ANOVA (**A**), 1-way ANOVA (**C** and **F**–**I**), and Student’s *t* test (**D**).

We first compared the anticancer effects of HITT in immune-competent BALB/c mice treated with anti-CD8α antibody to block CD8^+^ T cell cytotoxicity or the IgG control (Figure 1, A–C). As expected, murine mammary carcinoma 4T1 grew more quickly in mice treated with anti-CD8α antibody than in mice treated with IgG isotype control (Figure 1, A–C). HITT overexpression in 4T1 cells attenuated tumor growth under both conditions (Figure 1, A–C), but it suppressed tumor growth more evidently in the control mice (HITT/vector control: 25%–34%) than in anti-CD8α antibody–treated mice (HITT/vector control: 78%–80%) (Figure 1, A–C). This is not due to the different HITT fold changes (Figure 1D). In line with above data, MTT and BrdU incorporation assays revealed no obvious intrinsic impacts of HITT on cell viability and proliferation in 4T1 cells (Supplemental Figure 1, A and B; supplemental material available online with this article; https://doi.org/10.1172/JCI162951DS1). Because of this observation, the effects of HITT expression by cancer cells on T cell activity were further explored. MDA-231 (breast cancer) and HeLa (cervical cancer) cells stably expressing HITT and vector controls were successfully established and validated by quantitative reverse-transcription PCR (RT-PCR) (Supplemental Figure 1C). CD8^+^ T cells were isolated from human blood and activated as described previously (25) and then cocultured with the established cancer cell lines (Figure 1E). HITT overexpression by cancer cells elevated cytotoxic T lymphocyte (CTL) activity, as indicated by increased secretion of IL-2 and IFN-γ in the culture medium (Figure 1F). In agreement, HITT-overexpressing cells also exhibited increased vulnerability to CTL attack (Figure 1G). CRISPR/Cas–mediated HITT KO produced opposing results regarding both IL-2 and IFN-γ secretion and T cell–mediated cancer-killing effects (Figure 1, H and I, and Supplemental Figure 1D). Thus, HITT expression by cancer cells plays an important role in promoting T cell immunity.

### HITT inhibits PD-L1 expression.

**Figure 2:**
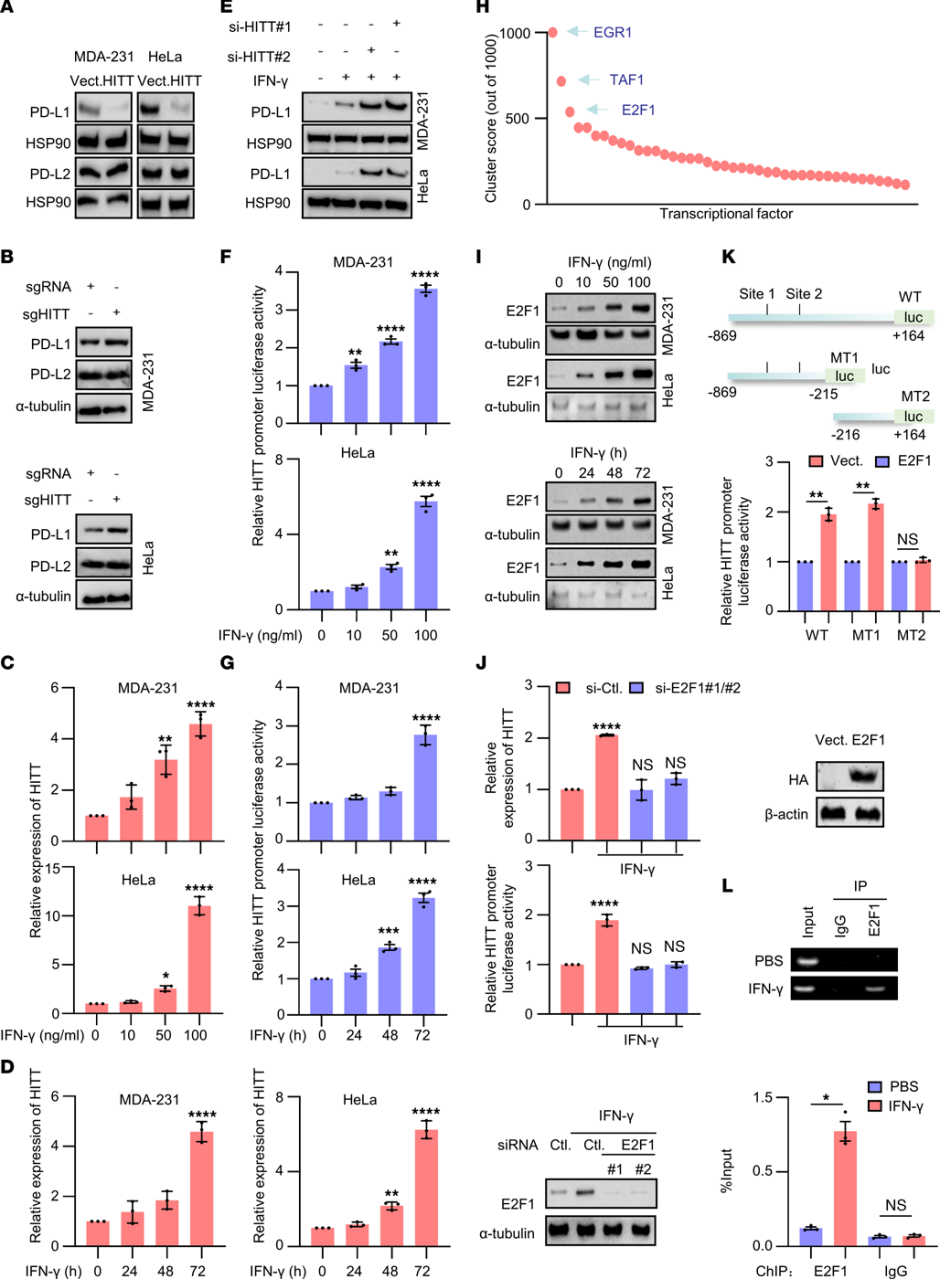
IFN-γ–induced and E2F1-mediated transactivation of HITT attenuates PD-L1 expression. (**A** and **B**) PD-L1 and PD-L2 protein levels analyzed by WB assay in HITT stable overexpression (**A**) or HITT-KO (**B**) cells. (**C** and **D**) HITT levels determined by qRT-PCR in MDA-231 and HeLa cells treated with different concentrations of IFN-γ for 24 hours (**C**) or treated for the indicated time periods with 10 ng/ml IFN-γ (**D**). (**E**) PD-L1 protein levels analyzed by WB in IFN-γ–treated cells with or without HITT KD. (**F** and **G**) HITT promoter luciferase activities determined by luciferase reporter assay in MDA-231 and HeLa cells treated with different concentrations of IFN-γ for 24 hours (**F**) or the indicated time periods with 10 ng/ml IFN-γ (**G**). (**H**) Relative binding potentials between different transcription factors and HITT promoter region were analyzed by UCSC ChIP sequence data. (**I**) E2F1 protein levels were detected by WB in MDA-231 and HeLa cells with different concentrations of IFN-γ for 24 hours or with 10 ng/ml IFN-γ for different time courses. (**J**) HITT expression levels and HITT promoter luciferase activities were measured by qRT-PCR and luciferase reporter assay in IFN-γ–treated (10 ng/ml for 24 hours) cells after E2F1 KD. E2F1 KD efficiency was validated by WB (bottom). (**K**) HITT promoter (full length and MT) controlled luciferase activities were determined after transient transfection of the indicated reporter plasmids together with E2F1 expression plasmid. (**L**) Binding between HITT promoter region and E2F1 was determined by ChIP assay after IFN-γ treatment (10 ng/ml for 24 hours). PCR band intensities were quantified using ImageJ and are presented in the bar graph (bottom). Data are derived from 3 independent experiments and are shown as mean ± SEM. *P < 0.05; **P < 0.01; ***P < 0.001; ****P < 0.0001; NS, not significant by 1-way ANOVA (**C**, **D**, **F**, **G**, and **J**) and Student’s *t* test (**K** and **L**).

To understand how HITT attenuates T cell immunity, we compared mass-spectrum data in the control and HITT knockdown (KD) HeLa cells. Unsupervised hierarchical-clustering analyses showed that the HITT-KO samples were clustered separately with the controls (Supplemental Figure 1E). A volcano plot demonstrates that 69 proteins were differentially regulated by HITT KO using a threshold of *P* ≤ 0.05 and fold change ≥ 1.8, with PD-L1 as one of the top hits (Supplemental Figure 1F). Therefore, the impacts of HITT on PD-L1 expression were explored. Remarkably, PD-L1 was dramatically reduced in HITT-overexpressing human breast cancer cells (MDA-231, MDA-468, and BT549), mouse mammary cancer cells (4T1), cervical cancer cells (HeLa), and colon cancer cells (HT29) (Figure 2A and Supplemental Figure 1G). In contrast, HITT KO or siRNA-mediated HITT KD led to increased PD-L1 expression (Figure 2B and Supplemental Figure 1G). Restoration of HITT expression abolished HITT KD–mediated PD-L1 elevation (Supplemental Figure 1H), while the expression of another family member, PD-L2, was unaffected (Figure 2, A and B). PD-L1 localization was not changed by HITT (Supplemental Figure 1I). Therefore, HITT mainly regulates PD-L1 by repressing its expression, but not by changing its localization.

Intriguingly, HITT expression was increased in a dose- and time-dependent manner in response to IFN-γ exposure in MDA-231 and HeLa cells (Figure 2, C and D). In addition, IFN-γ–induced HITT expression was relatively common because treatment led to increased HITT expression in all breast cancer cell lines tested regardless of their genetic features (Supplemental Figure 2A and Supplemental Table 1). IFN-γ–induced HITT expression was also observed in lung cancer cells, such as H23 and H1299 (Supplemental Figure 2A). These data suggest that HITT is a newly identified IFN-γ signal–responsive lncRNA. In addition, we observed that PD-L1 expression was increased by IFN-γ, whereas 2 independent siRNA-mediated HITT KDs augmented IFN-γ–induced PD-L1 expression (Figure 2E). Therefore, HITT plays important roles in attenuating PD-L1 expression under both basal and IFN-γ–stimulated conditions.

### E2F1 transactivates HITT upon IFN-γ stimulation.

Given the essential role of HITT in regulating PD-L1 expression, we further explored the underlying mechanisms of IFN-γ–induced HITT expression. HITT promoter luciferase reporter and luciferase-HITT reporter were generated (Supplemental Figure 2B). HITT promoter–driven luciferase activity was elevated in a dose- and time-dependent manner following IFN-γ treatment (Figure 2, F and G), while luciferase-HITT reporter activity was unchanged under the same conditions (Supplemental Figure 2C), suggesting that HITT is activated by IFN-γ at the transcriptional level. In line with these results, actinomycin D (ActD), an mRNA synthesis inhibitor, abolished IFN-γ–induced HITT expression (Supplemental Figure 2D).

We then analyzed the UCSC Genome Browser ChIP-sequencing database (Figure 2H). The most potent transcription factors were early growth response 1 (EGR1), TATA-box binding protein associated factor 1 (TAF1), and E2F transcription factor 1 (E2F1) (Figure 2H). IFN-γ treatment barely affected the expression of EGR1 (Supplemental Figure 2E). Despite detection of increased levels of TAF1 in a time-dependent manner after IFN-γ treatment, diminishing its expression by siRNA failed to influence HITT levels (Supplemental Figure 2F). In contrast, E2F1 was remarkably enhanced by IFN-γ in a dose- and time-dependent manner, accompanied by a coordinate increase of HITT expression (Figure 2I). Inhibition of E2F1 expression by 2 independent small interfering E2F1s (si-E2F1s) completely abolished IFN-γ–induced HITT expression and HITT promoter luciferase activity (Figure 2J).

In addition, ectopic E2F1 expression increased HITT levels and HITT promoter–driven luciferase activity in an E2F1 dose–dependent manner (Supplemental Figure 2G), while KD of endogenous E2F1 reduced them (Supplemental Figure 2H). Furthermore, the activity of mutant type 1 (MT1) luciferase reporter, which contains the predicted E2F1-binding sites, was as effective as that of WT reporter in response to E2F1 expression (Figure 2K), whereas MT2 luciferase reporter, without the predicted binding motif, largely lost its response to E2F1. Moreover, binding between E2F1 and the HITT promoter region was verified by a ChIP assay, and binding was increased after IFN-γ treatment (Figure 2L). E2F1 is therefore required for transcriptional activation of its target HITT upon IFN-γ stimulation.

### HITT and RGS2 coordinately inhibit PD-L1 translation.

**Figure 3:**
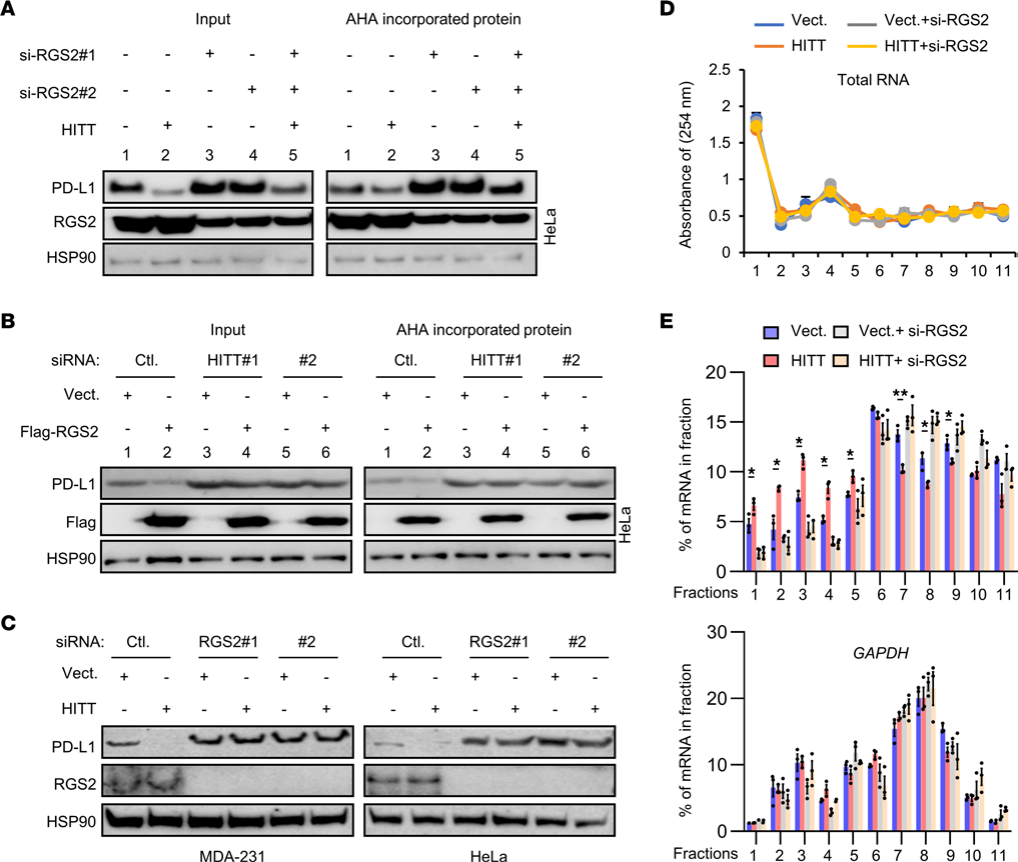
HITT inhibits PD-L1 translation in an RGS2-dependent manner. (**A** and **B**) Affinity purification of biotinylated AHA-labeled acutely synthesized proteins of PD-L1, RGS2, and HSP90 was detected by WB after HITT overexpression with or without RGS2 KD (**A**) or RGS2 overexpression with or without HITT KD (**B**). (**C**) PD-L1 protein levels were analyzed by WB in HITT stable lines with or without RGS2 KD. (**D** and **E**) Polysome in the cytoplasm was fractionated through sucrose gradients. The total RNA amount was determined by the intensity at 254 nm (**D**), and PD-L1 and GAPDH mRNA levels were detected by qRT-PCR (**E**) in gradient fractions of HITT stable-expression HeLa cells with or without RGS2 KD. Representative data as a percentage of total RNA of interest in the gradient from 3 independent experiments are presented. *P < 0.05; **P < 0.01, Student’s *t* test (**D** and **E**).

Meanwhile, considering the essential role of PD-L1 in immune evasion, we investigated the mechanisms underlying HITT-inhibited PD-L1 expression. First, we found no obvious change in the expression of *Cd274* mRNA, encoding for PD-L1, after HITT overexpression or KD (Supplemental Figure 3, A and B). Secondly, neither lysosome inhibitor chloroquine nor proteasome inhibitor MG132 influenced HITT-mediated PD-L1 inhibition (Supplemental Figure 3, C and D). Intriguingly, a click chemistry and l-azidohomoalanine–label (AHA-label) assay revealed that HITT overexpression inhibited newly synthesized PD-L1 protein (Figure 3A), while HITT KD promoted it (Figure 3B), with the newly synthesized HSP90 serving as a negative control (Figure 3, A and B).

It is reasonable to suppose that HITT may fulfil its roles by cooperating with translational regulators. To test this hypothesis, we first utilized the Gene Ontology (GO) database to search translational regulators in the genome. In total, 78 proteins were identified as negatively involved in protein translation. Among them, we identified 15 proteins that have been reported to be directly or indirectly related to T cell immunity via a literature search (Supplemental Table 2). We then used RNA interference techniques to specifically inhibit the expression of those individual genes (Supplemental Figure 4A). KD efficiency was verified in each case by qRT-PCR. Western blot (WB) assay revealed an obvious increase of PD-L1 protein expression in the (RGS2) KD cells, but not others (Figure 3C and Supplemental Figure 4A). Intriguingly, the ability of HITT to regulate PD-L1 expression was largely diminished by RGS2 KD (Figure 3C). RGS2 had little effect on PD-L1 expression on the mouse cell line 4T1, which does not contain HITT, and overexpression of HITT in 4T1 cells restored the effects of RGS2 KD on PD-L1 expression (Supplemental Figure 4B). Furthermore, the click chemistry and AHA-label assay showed that RGS2 KD increased the levels of the newly synthesised PD-L1 protein and also abolished HITT overexpression–inhibited PD-L1 expression (Figure 3A). In contrast, RGS2 overexpression repressed the newly synthesized PD-L1 protein and also rescued HITT KD–induced PD-L1 expression (Figure 3B). Coordinated regulation of PD-L1 translation by RGS2 and HITT was further validated by a chromosome fractionation assay (Figure 3, D and E). Namely, RGS2 and HITT similarly reduced polysome-occupied *Cd274* mRNA and no further reduction was observed with their combination (Figure 3E). These data suggest that HITT and RGS2 coordinately regulate PD-L1 translation through the same mechanism.

### 1,080-1,130 nt HITT is physically associated with F194, Q196, and D197 in the RGS domain of RGS2.

**Figure 4:**
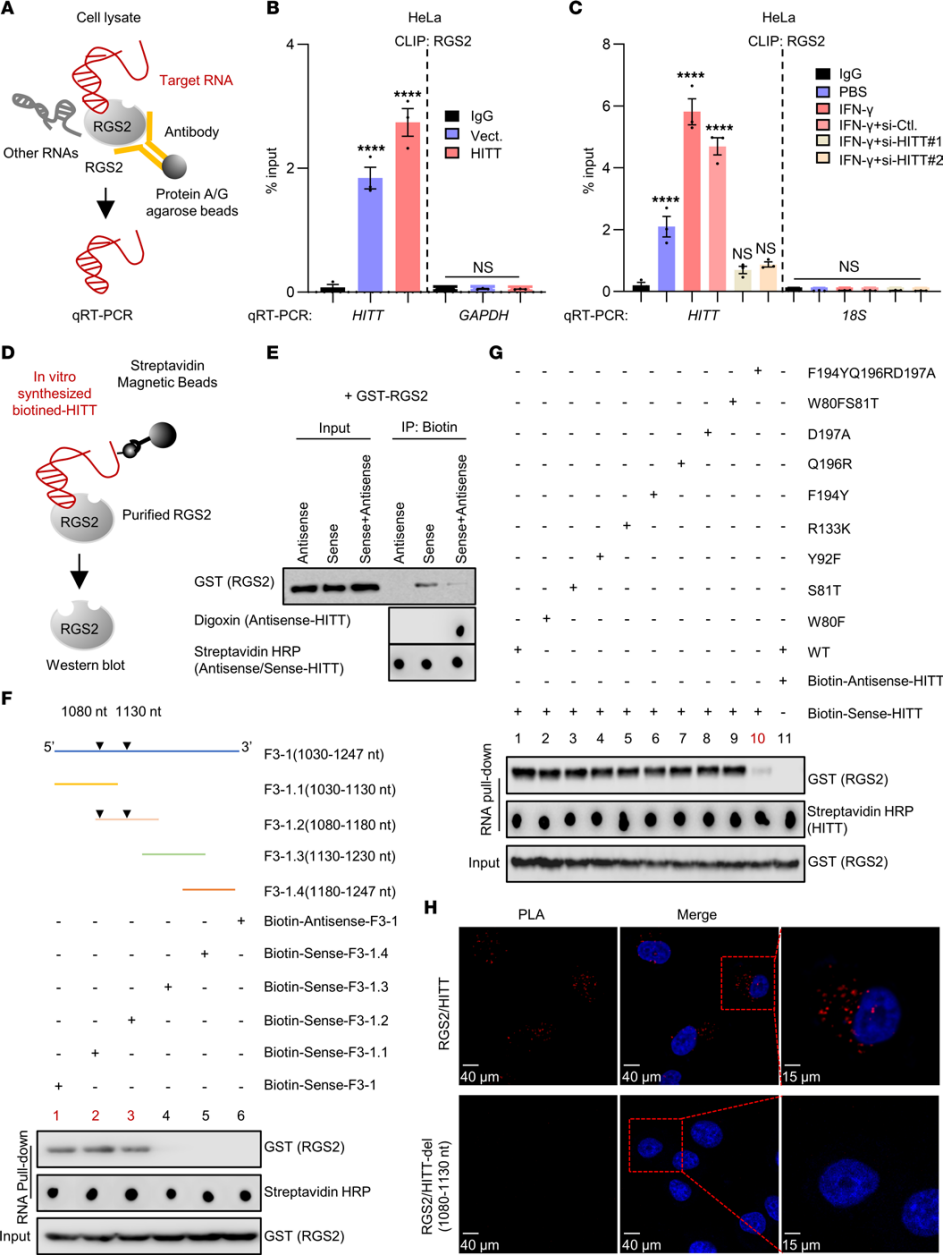
RGS2 is a binding partner of HITT. (**A**) Schematic of CLIP assay for binding between RGS2 and HITT in living cells. (**B** and **C**) HITT levels determined by qRT-PCR following CLIP RGS2 after HITT overexpression (**B**) or KD in the presence or absence of IFN-γ treatment (**C**) in HeLa cells, with GAPDH or 18s mRNA and CLIP IgG as negative controls. (**D**) Schematic of in vitro RNA pull-down assay to analyze the binding between in vitro–synthesized biotin-labeled HITT and purified RGS2. (**E**) GST-tagged RGS2 protein coprecipitated with biotin-sense-HITT in the presence or absence of digoxin-antisense-HITT. (**F**) RGS2 protein coprecipitated by biotin-HITT-F3-1 (1,030–1,247 nt) or its fragments determined by RNA pull-down assay. Schematic showing sequentially fragmented HITT-F3-1 (1,030–1,247 nt). (**G**) GST-tagged full-length RGS2 or its mutants coprecipitated with biotin-sense-HITT determined by WB. (**H**) PLA analysis of endogenous RGS2/exogenous HITT or HITT-del (1,080–1,130 nt) in HeLa cells. Data derived from 3 independent experiments are presented as mean ± SEM in the bar graph. ****P < 0.0001; NS, not significant by 1-way ANOVA (**B** and **C**). Scale bars: 40 μm (left and center panels); 15 μm (right panels).

Given their coordinated effects on PD-L1 translation, we speculated that HITT may bind with RGS2. Indeed, a UV cross-linking and immunoprecipitation (CLIP) assay (Figure 4A) revealed that HITT and RGS2 physically associate with each other in living cells, and their association was increased after ectopic HITT overexpression (Figure 4B). Consistently, their binding was increased by IFN-γ, while inhibition of IFN-γ–induced HITT expression by si-HITT abolished such an effect (Figure 4C and Supplemental Figure 4C). Direct binding between HITT and RGS2 was also validated by RNA pull-down assay using in vitro–synthesised biotinylated HITT and purified RGS2 protein, and their binding was suppressed by antisense HITT (Figure 4, D and E).

The key RGS2-binding region in HITT was initially mapped to F3-1 (1,030–1,247 nt) by in vitro binding assay (Supplemental Figure 5A). After that, this fragment was sequentially truncated to 4,100 nt fragments with 50 nt sequence overlap (F3-1.1~4, Figure 4F). Among those, F3-1.1 (1,030–1,130 nt) and F3-1.2 (1,080-1,180 nt) bound with RGS2 to similar extents, suggesting that their overlapping region mapped to 1,080–1,130 nt contains the key nucleotides in binding RGS2 (Figure 4F). No other HITT F3-1 fragmented mutants (F3-1.3 and F3-1.4) were found to bind with RGS2 (Figure 4F).

By mixing truncated RGS2 protein with HITT, we found that C-terminal RGS2 (80–212 aa), containing the RGS domain, is necessary for its binding with HITT (Supplemental Figure 5B). We further identified the most potential residues by analysis of the top 10 RGS2-HITT (1,080–1,130 nt) models predicted by HDOCK (26). Seven RGS2 residues (W80, S81, Y92, R133, F194, Q196, and D197) were identified as the most potentially binding sites in bridging their interaction because they were predicted by these 10 models for at least 5 times and with a root mean square deviation (RMSD) value of less than 3Å (Supplemental Table 3). Then, each of these amino acids was substituted (W80F, S81T, Y92F, R133K, F194Y, Q196R, and D197A), and the combined substitution was generated (W80FS81T and F194YQ196RD197A) when they were close or next to each other (Supplemental Figure 5C). The following RNA pull-down assay revealed that none of the single substitutions had impact on the interaction between RGS2 and HITT (1,080–1,130 nt). However, their interaction was largely diminished by triple mutation at site F194YQ196RD197A (Figure 4G), suggesting that F194, Q196, and D197 form the surface to interact with HITT. The direct interaction between RGS2 and HITT was verified using the proximity ligation assay (PLA) in cells transfected with HITT, but not those transfected with RGS2 binding defective mutant HITT-del (1,080–1,130 nt) (Figure 4H). Thus, HITT directly binds with RGS2 mainly at F194, Q196, and D197 via its (1,080–1,130 nt) fragment. The interaction may be essential for their regulation of PD-L1 (see below).

### K175, R176, and S179 in RGS domain are required for PD-L1–5′-UTR binding.

**Figure 5:**
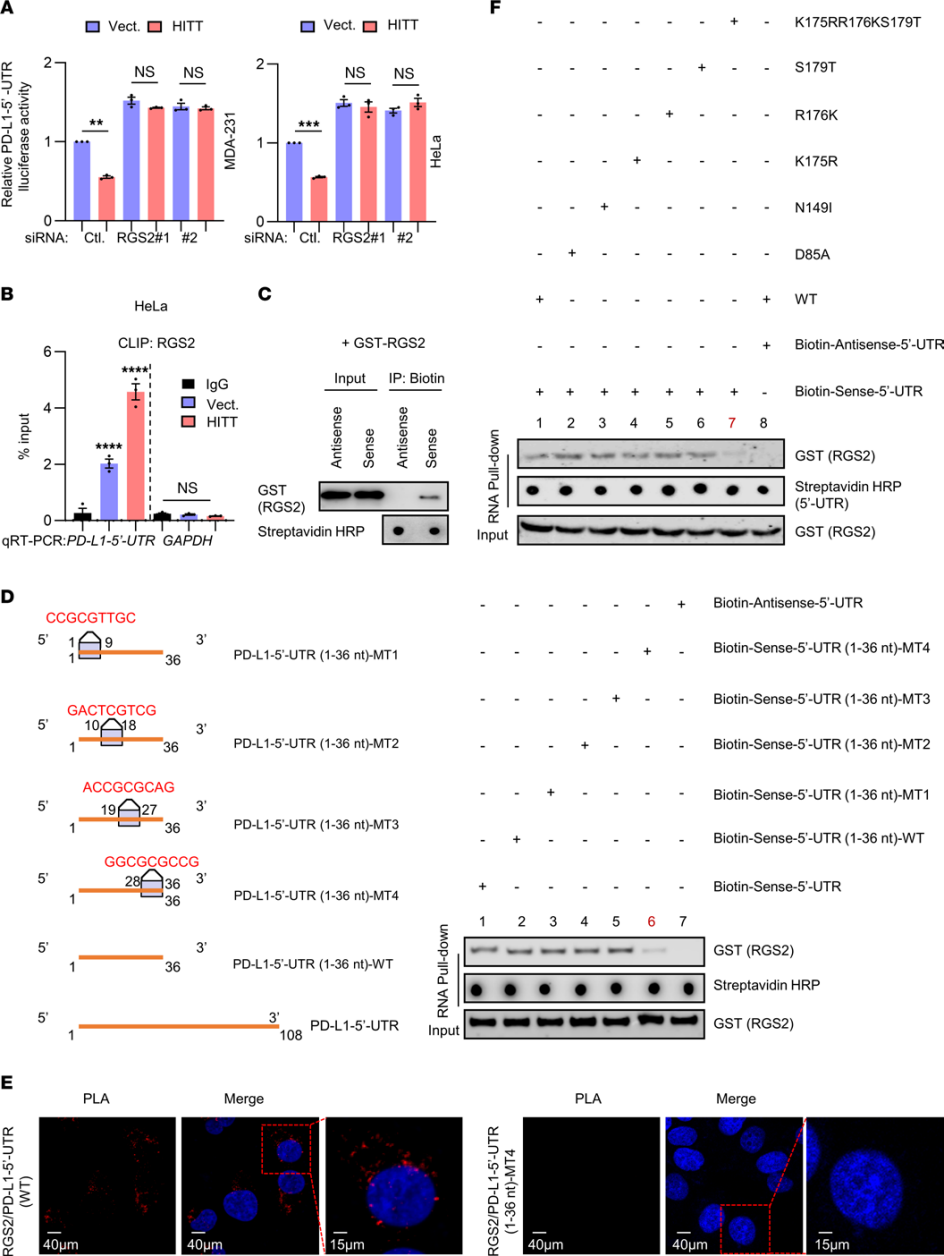
RGS2 physically binds with PD-L1–5′-UTR. (**A**) PD-L1–5′-UTR–driven luciferase activities determined in HITT stable lines with or without RGS2 KD. (**B**) PD-L1–5′-UTR levels determined by qRT-PCR following CLIP RGS2 in HITT-overexpressing stable HeLa cells, with GAPDH mRNA and CLIP IgG as negative controls. (**C**) GST-tagged RGS2 protein coprecipitated with biotin–PD-L1–5′-UTR or biotin-PD-L1–5′-UTR antisense control determined by WB. (**D**) Schematic of the compensatory mutations in PD-L1–5′-UTR (1–36 nt). GST-tagged RGS2 protein coprecipitated with biotin-PD-L1–5′-UTR (1–36 nt) or its mutants, determined by RNA pull-down assay. (**E**) PLA analysis of endogenous RGS2/exogenous PD-L1–5′-UTR or 5′-UTR (1-36 nt) MT4 in HeLa cells. (**F**) GST-tagged RGS2 or mutant proteins coprecipitated with biotin–PD-L1–5′-UTR (1–36 nt) determined by RNA pull-down assay. Data derived from 3 independent experiments are presented as mean ± SEM. **P < 0.01; ***P < 0.001; ****P < 0.0001; NS, not significant by Student’s *t* test (**A**) and 1-way ANOVA (**B**). Scale bars: 40 μm (left and center panels); 15 μm (right panels).

We next asked how the RGS2/HITT complex influences PD-L1 translation. To answer this question, we generated 2 luciferase reporter plasmids, *PD-L1–5*′*-*UTR and *3*′*-*UTR luciferase reporters (as shown in the diagram, Supplemental Figure 5D). Strikingly, it was with *PD-L1–5*′-UTR, but not *PD-L1*–*3*′-UTR, that luciferase reporter activity was decreased by HITT overexpression and increased by HITT KD (Supplemental Figure 5, E and F). RGS2 KD enhanced *PD-L1–5*′-UTR luciferase activity and completely abolished the effect of HITT (Figure 5A), confirming that RGS2/HITT imparts negative regulation of PD-L1 expression through the 5′-UTR.

We further explored how RGS2/HITT regulates *PD-L1–5*′-UTR–dependent PD-L1 expression. It has been proposed before that RGS2 inhibits protein translation by binding with eIF2Bε (16). However, this is unlikely for RGS2-regulated PD-L1 expression (Supplemental Figure 5G). Intriguingly, by using a CLIP assay and RNA pull-down assay, as indicated in Figure 4, A and D, we found that RGS2 not only served as a HITT-binding protein as described above (Figure 4, B and E), but also associated with the *PD-L1–5*′-UTR both in living cells and in vitro (Figure 5, B and C). The extreme 5′ end (1–36 nt) in the *PD-L1–5*′-UTR is essential for RGS2 binding because the 1–36 nt and 1–72 nt regions, but not 37–108 nt, in the *PD-L1–5*′-UTR were found to coprecipitate with RGS2 (Supplemental Figure 5H). We then generated 4 compensatory mutants spanning across 1–36 nt *PD-L1–5*′-UTR, as depicted in Figure 5D. Intriguingly, when 28–36 nt were substituted with their compensatory sequences (MT4), *PD-L1–5*′-UTR (1–36 nt) lost its RGS2-binding ability (Figure 5D), suggesting that the intact 28–36 nt is required for *PD-L1–5*′-UTR’s interaction with RGS2. Consistently, PLA-positive RGS2/*PD–L1-5*′-UTR complexes, but not RGS2/*PD-L1–5*′-UTR 1–36 nt MT4 complexes, were detected in HeLa cells (Figure 5E).

We also mapped the key *PD-L1–5*′-UTR–binding residues in RGS2. Similarly to HITT, *PD-L1–5*′-UTR also bound to RGS2 (80–212 aa), as revealed by the in vitro RNA-binding assay (Supplemental Figure 5I). Following approaches similar to those described in Figure 4G, we predicted a set of residues, D85, N149, K175, R176, and S179, that may mediate RGS2’s binding with *PD-L1–5*′-UTR using HDOCK (Supplemental Figure 5J and Supplemental Table 3). We tested the binding ability of the single mutants at each of these sites or triple-mutant K175RR176KS179T (Figure 5F) and found that K175RR176KS179T remarkably reduced its binding with *PD-L1–5*′-UTR. Therefore K175, R176, and S179 provide the major *PD-L1–5*′-UTR–binding sites of RGS2 (Figure 5F).

### HITT forms an RNA-RNA duplex with the PD-L1–5′-UTR.

**Figure 6:**
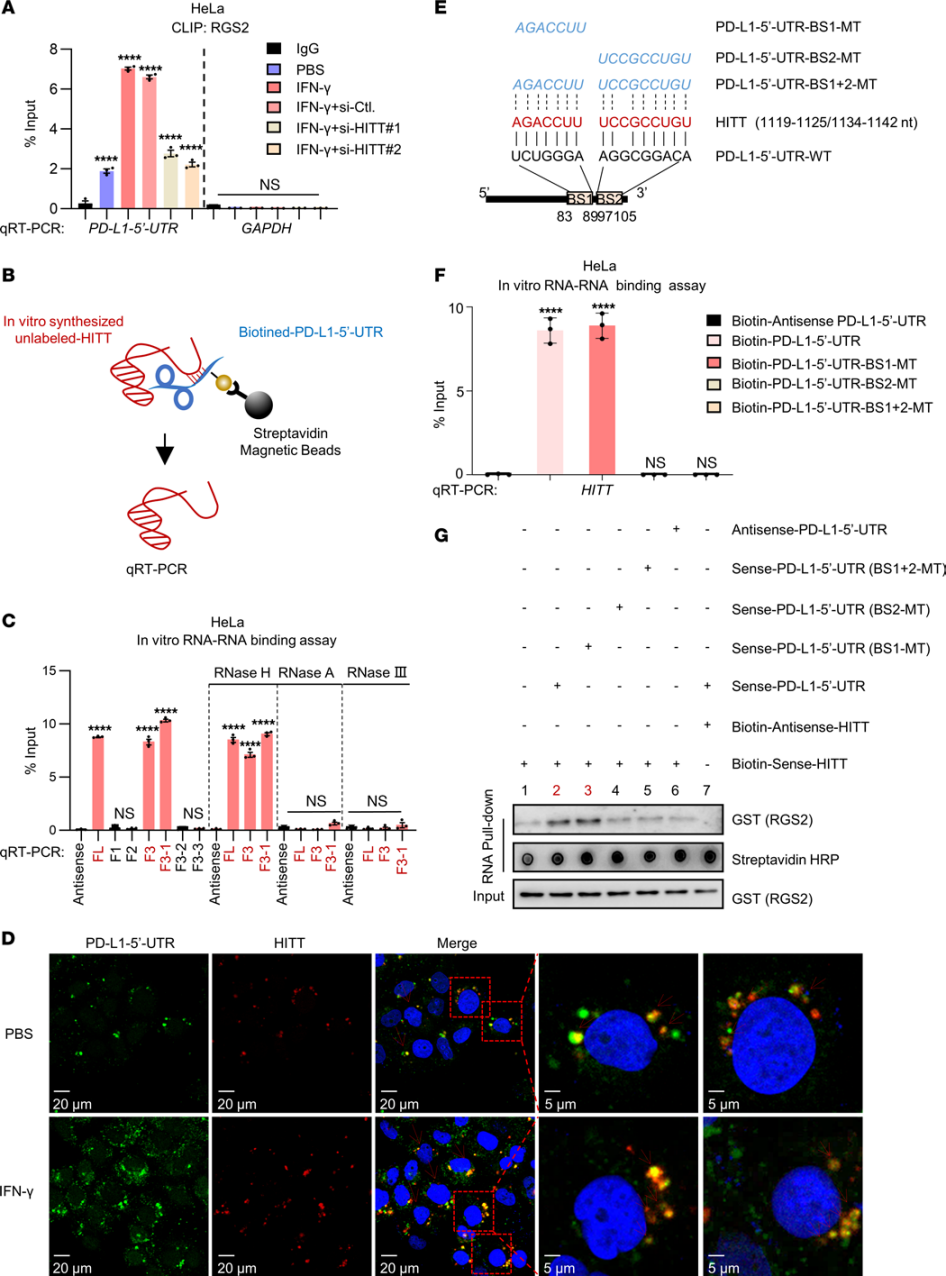
HITT forms RNA-RNA duplex with *PD-L1–5*′-UTR. (**A**) PD-L1–5′-UTR levels determined by qRT-PCR following CLIP RGS2 under IFN-γ treatment with or without HITT KD, with GAPDH mRNA and CLIP IgG as negative controls. (**B**) Schematic showing in vitro RNA-RNA binding assay to detect the binding between in vitro–synthesized unlabeled HITT and biotin–PD-L1–5′-UTR. (**C**) HITT and HITT fragments pulled down by biotin–PD-L1–5′-UTR, biotin-PD-L1–5′-UTR fragments, or biotin–antisense–PD-L1–5′-UTR control determined by qRT-PCR with or without RNase H, RNase A, or RNase III. (**D**) FISH showing colocalization between HITT and PD–L1–5′-UTR in PBS or IFN-γ–treated HeLa cells. (**E**) Schematic showing complementary sequence (BSs) between HITT and PD-L1–5′-UTR according to the prediction of an online bioinformatic tool (http://rna.informatik.uni-freiburg.de/IntaRNA/Input.jsp). Three PD-L1–5′-UTR mutations, which lost the complementarity site of PD-L1–5′-UTR at BS1 (BS1-MT), BS2 (BS2-MT), and both BS1 and BS2 (BS1+2-MT) were generated and are shown in the diagram. (**F**) HITT coprecipitated by biotin–PD-L1–5′-UTR (WT or mutants) or biotin–antisense–PD-L1–5′-UTR control determined by qRT-PCR. (**G**) GST-tagged RGS2 pulled down by biotin-HITT and biotin-antisense-HITT control in the presence of unlabeled FL PD-L1–5′-UTR or PD-L1–5′-UTR mutants determined by WB in an in vitro RNA pull-down assay. Data derived from 3 independent experiments are presented as mean ± SEM. ****P < 0.0001; NS, not significant by 1-way ANOVA (**A**, **C**, and **F**). Scale bars: 20 μm (left 3 panels); 5 μm (right 2 panels).

The newly identified binding mechanisms of RGS2/HITT and RGS2/*PD-L1–5*′-UTR and the coordinated inhibitory effect of HITT and RGS2 on PD-L1 translation inspired us to explore how HITT contributes to RGS2-regulated and 5′-UTR–dependent PD-L1 translation. To this end, we first compared the binding of RGS2/*PD-L1–5*′-UTR in cells with different expression levels of HITT. The results showed that IFN-γ elevated HITT expression, which was accompanied by increased RGS2/*PD-L1–5*′-UTR binding (Figure 6A and Supplemental Figure 4C), while inhibition of IFN-γ–induced HITT expression dramatically reduced RGS2/*PD-L1–5*′-UTR complex levels (Figure 6A). Arbitrarily, expression of HITT produced an effect similar to that of IFN-γ–mediated endogenous HITT overexpression (Figure 6A). These data suggest that HITT facilitates binding between RGS2 and *PD-L1–5*′-UTR.

We further explored how HITT fulfills such a task by testing whether it forms an RNA-RNA complex with *PD-L1–5*′-UTR. In this RNA-RNA binding assay (27), we found that in vitro–synthesised HITT (unlabeled) was associated with biotin-labeled *PD-L1–5*′-UTR, but not biotin-labeled antisense *PD-L1–5*′-UTR (Figure 6, B and C). Remarkably, HITT antisense RNA disrupted the binding between HITT and *PD-L1–5*′-UTR (Supplemental Figure 6A). In addition, their binding was completely abrogated by RNase III or RNase A, but not RNase H (Figure 6C), suggesting the double-stranded RNA (HITT/PD-L1–5′-UTR) is formed. Furthermore, the colonization of HITT/*PD-L1–5*′-UTR was detected by FISH using Cy3-labeled HITT probe and FAM-labeled PD-L1–5′-UTR probe in cells under both basal and IFN-γ–treated conditions (Figure 6D).

The RNA-RNA binding assay also revealed that HITT F3 (1,030–2,050 nt) and F3-1 (1,030–1,247 nt), but not other mutant fragments, contributed to *PD-L1–5*′-UTR binding (Figure 6C). The binding motif between F3-1 (1,030–1,247 nt) and *PD-L1–5*′-UTR was further analyzed using an RNA-RNA interaction bioinformatic tool, IntaRNA. The highest potential binding site between 2 RNA molecules was predicted to be 83–89 nt (binding site 1 [BS1]) and 97-105 nt (BS2) in *PD-L1–5*′-UTR (Figure 6E). To validate this bioinformatic result, point mutations on the *PD-L1–5*′-UTR that aimed to disrupt the RNA-RNA duplex were synthesized, as shown in Figure 6E. No binding was detected between HITT and the biotin-labeled BS2-MT and BS1+2-MT *PD-L1–5*′-UTRs in the in vitro binding assay (Figure 6F), whereas WT and BS1-MT *PD-L1–5*′-UTRs, both of which retained the ability to bind with HITT, were found to dramatically improve RGS2’s binding with the streptavidin magnetic beads to pull down biotin-HITT. However, the BS2-MT and BS1+2-MT PD-L1–5′-UTRs, the 2 HITT binding-defective mutants, failed to do so (Figure 6G). Neither BS1 nor BS2 influenced *PD-L1–5*′-UTR’s binding with RGS2 (Supplemental Figure 6B), which is consistent with above data showing that 1–36 nt is essential for *PD-L1–5*′-UTR/RGS2 binding (Supplemental Figure 5H). In addition, HITT strengthened the binding between RGS2 and PD-L1–5′-UTR-WT or BS1-MT, but not the binding between RGS2 and PD-L1–5′-UTR-BS2-MT or BS1+2-MT (Supplemental Figure 6B). These data show that HITT bridges and strengthens the interaction of *PD-L1–5*′-UTR with RGS2 by direct interaction with *PD-L1–5*′-UTR at BS2 (Supplemental Figure 6C).

### HITT/PD-L1–5′-UTR/RGS2 interactions are essential for PD-L1 inhibition.

**Figure 7:**
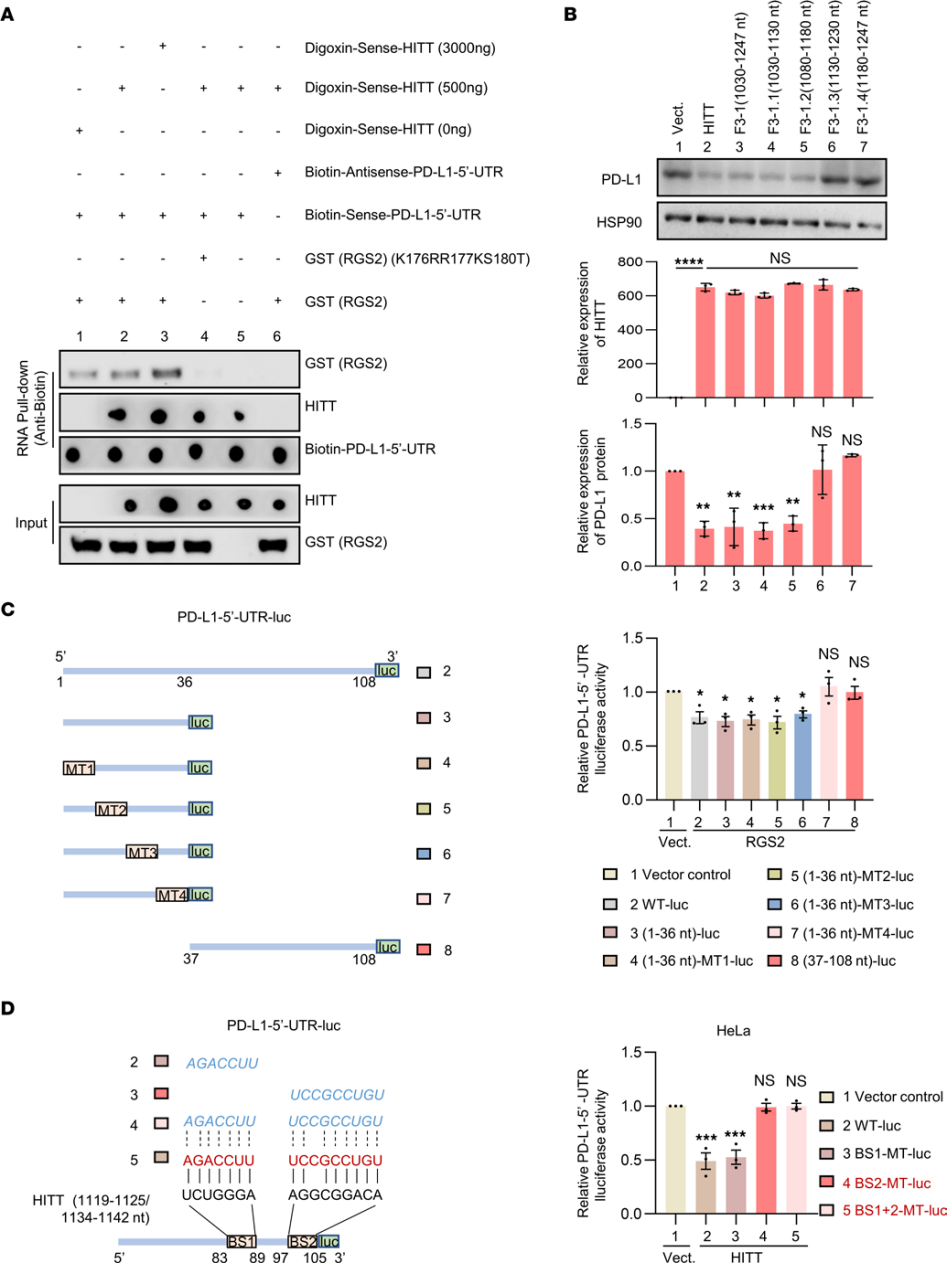
RGS2, HITT, and PD-L1–5′-UTR interaction is required for PD-L1 inhibition. (**A**) The interactions between RGS2, HITT, and PD-L1–5′-UTR RNA determined by RNA pull-down assays. (**B**) PD-L1 protein levels after transfection with HITT, F3-1, F3-1.1, F3-1.2, F3-1.3, and F3-1.4 into HeLa cells. HITT and its mutant overexpression efficiencies were measured by qRT-PCR, and PD-L1 intensities were quantified and are shown in bar graph. (**C** and **D**) Reporter activities of the indicated luciferase reporters before and after RGS2 overexpression (**C**) or HITT overexpression (**D**). Data derived from 3 independent experiments are presented as mean ± SEM.*P < 0.05; **P < 0.01; ***P < 0.001; ****P < 0.0001; NS, not significant by 1-way ANOVA test (**B**–**D**).

To validate a model where 3 molecules interact to inhibit PD-L1 translation, anti-biotin–conjugated beads were used to pull down biotin-labeled PD-L1–5′-UTR and its possible binding partners in the mixture. As shown, coprecipitated HITT was gradually increased with rising doses of digoxin-labeled HITT in the mixture (Figure 7A). Intriguingly, despite the same amount of RGS2 protein in the mixture, its binding with PD-L1–5′-UTR was also gradually increased with rising doses of HITT (Figure 7A). Therefore, the increased HITT not only enhances its own binding with PD-L1–5′-UTR, but also facilitates the binding of RGS2 with PD-L1–5′-UTR, suggesting the 3 molecules form one complex. We also found that HITT lost its ability to improve the binding between PD-L1–5′-UTR and PD-L1–5′-UTR binding–deficient RGS2 (K175RR176KS179T) (Figure 7A), suggesting that HITT recruits RGS2 to the complex and also promotes direct binding between RGS2 and PD-L1–5′-UTR (Supplemental Figure 6C).

We then tested the essential roles of their interaction in regulating PD-L1 expression. First, the impact of the binding of RGS2 with HITT or PD-L1–5′-UTR was tested after overexpression of RGS2 WT, RNA-binding defective mutants (M2, K175RR176KS179T and M2, 194YQ196RD197A) and the combined mutant (M3, K175RR176KS179T-194YQ196RD197A) in HeLa cells. The expression of PD-L1 was examined by WB. The HITT or PD-L1–5′-UTR–binding defective mutants repressed PD-L1 expression, despite a relatively low efficiency when compared with WT RGS2 (Supplemental Figure 6D), whereas the combined substitution of all 6 amino acids completely abolished RGS2’s ability to inhibit PD-L1 (Supplemental Figure 6D). These data suggest that both bindings (RGS2/HITT and RGS2/PD-L1–5′-UTR) are essential for RGS2-mediated PD-L1 inhibition.

Second, the essential roles of HITT-mediated RGS2 binding were validated by another assay. As shown in Figure 7B, the fragments containing 1,080–1,130 nt HITT, such as full-length HITT, F3-1, F3-1.1, and F3-1.2, were able to inhibit PD-L1 expression (Figure 7B). The other fragments (F3-1.3 and F3-1.4) failed to do so (Figure 7B), further suggesting that the physical interaction between HITT and RGS2 is required for HITT-regulated PD-L1 inhibition.

Third, using luciferase reporter assays, we found that RGS2 binding defective mutant PD-L1–5′-UTR-MT4 (compensatory mutation at 28–36 nt), but not the other mutant reporter, failed to respond to RGS2 overexpression (Figure 7C), which provides additional evidence that RGS2/PD-L1–5′-UTR binding is essential for RGS2-mediated PD-L1 inhibition.

Fourth, the critical roles of HITT/PD-L1–5′-UTR interactions in regulating PD-L1 expression were also examined. We found that HITT inhibited the activities of PD-L1–5′-UTR luciferase reporters with intact HITT BS2, such as WT and PD-L1–5′-UTR-BS1-MT reporter, and failed to change the luciferase reporter activities of PD-L1–5′-UTR-BS2-MT or BS1+2-MT (Figure 7D). These data suggest that the intact HITT BS2 is necessary for HITT-mediated PD-L1 inhibition. These data show that the 3-way interaction among HITT, PD-L1–5′-UTR, and RGS2 is critical for the inhibition of PD-L1 translation.

### HITT inhibits T cell immunity in a PD-L1–dependent manner.

**Figure 8:**
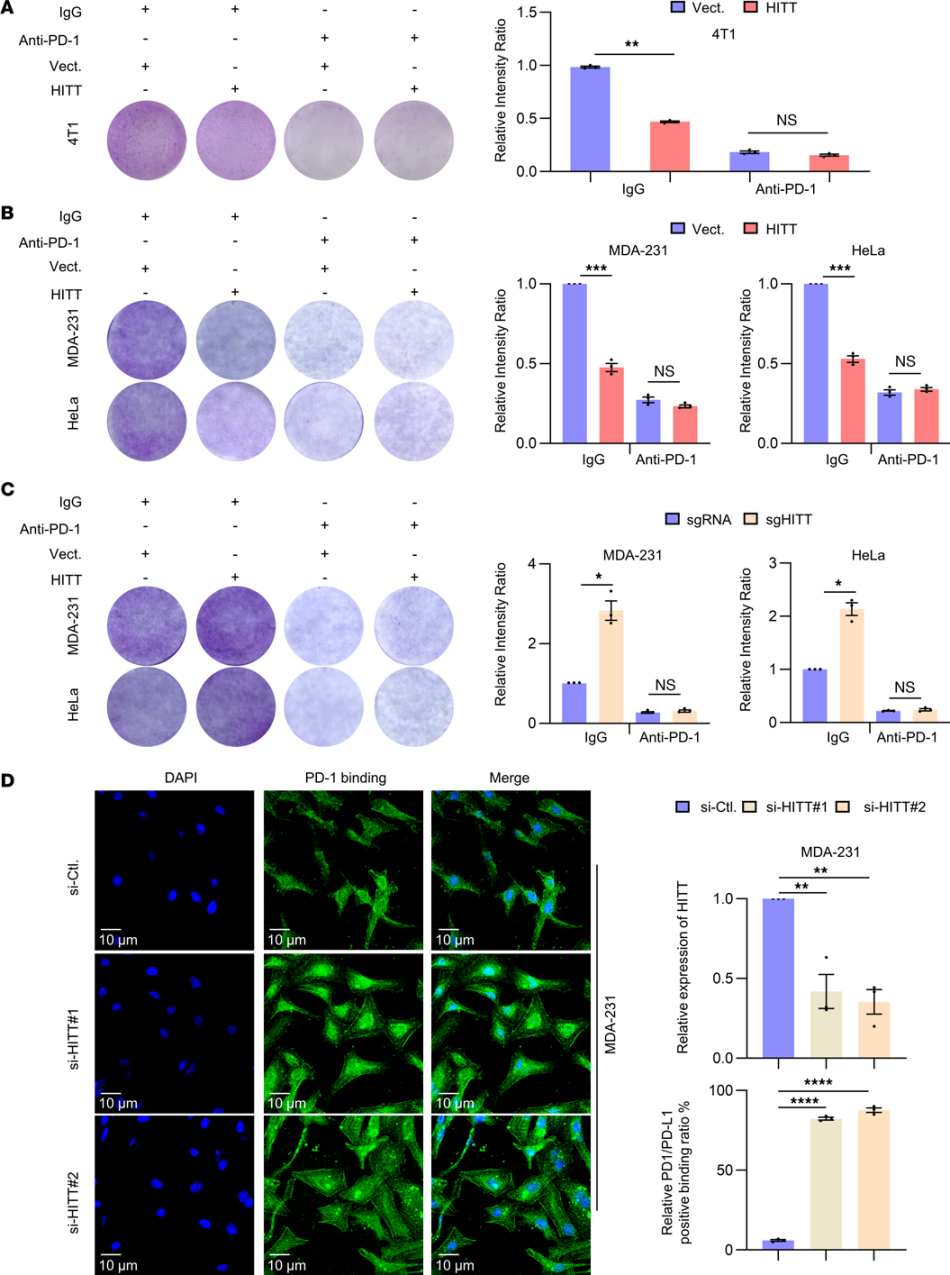
HITT enhances T cell–mediated tumor cell–killing efficacy in a PD-L1–dependent manner. (**A**) Detection of the attached 4T1-OVA cells by crystal violet staining after coculture with the activated mouse OT-I T cells for 2 days in the presence of anti–PD-1 antibody or IgG control. Intensities are shown in bar graph. (**B** and **C**) Detection of the attached MDA-231 and HeLa cells by crystal violet staining after coculture with the activated T cells for 6 hours in the presence of anti–PD-1 antibody or IgG control. Intensities are shown in bar graphs. (**D**) Immunostaining of PD-1 (fused to Ig-Fc) on HITT KD MDA-231 cells. PD-L1 fluorescence intensities at cell edge were quantified, and relative levels are shown in bar graph (right). HITT KD efficiency was determined by qRT-PCR. Data derived from 3 independent experiments are presented as mean ± SEM. *P < 0.05; **P < 0.01; *** *P* < 0.001; **** *P* < 0.0001; NS, not significant by Student’s *t* test (**A**–**C**) and 1-way ANOVA (**D**). Scale bars: 10 μm.

Given the essential role of HITT in inhibiting PD-L1 expression, we compared the killing effects of CTLs before and after blocking PD-L1 signaling via anti–PD-1 antibody in foreign antigen chicken OVA-expressing 4T1 cells (4T1-OVA). We consistently detected an increased killing effect of OT-I T cells after coculture with HITT-overexpressing 4T1-OVA cells (Figure 8A). Anti–PD-1 antibody increased the killing effect of CTLs, as reported previously (28). The HITT-regulated CTL killing effect was completely abrogated by blocking PD-L1 signaling (Figure 8A). Consistently, a similar effect of HITT on the killing effect of human CTLs after coculture with HITT overexpressing MDA-231 and HeLa cells was observed (Figure 8B and Supplemental Figure 7, A and B). Anti–PD-1 antibody or PD-L1 KD increased the killing effect of CTLs. The HITT-regulated CTL killing effect was completely abrogated by blocking PD-L1 signaling (Figure 8, B and C, and Supplemental Figure 7, A and B). In contrast, PD-L1 overexpression repressed CTL-mediated cancer cell killing effects, and it also abolished HITT-induced killing effects of CTL (Supplemental Figure 7C). In line with these data, HITT lost its ability to regulate expression levels of IL-2 and IFN-γ after anti–PD-1 treatment (Supplemental Figure 7D). These data demonstrate that HITT mainly regulates T cell immunity by suppressing PD-L1 expression. Consistently, HITT KD increased the binding of PD-1 protein to the surfaces of cancer cells, as shown in a PD-1–binding assay (Figure 8D). Thus, HITT markedly enhances T cell cytotoxicity by inhibiting PD-L1 expression in cancer cells, leading to reduced interaction between PD-L1 and PD-1.

### HITT inhibits tumor growth in vivo by preventing PD-L1–mediated T cell deactivation.

**Figure 9:**
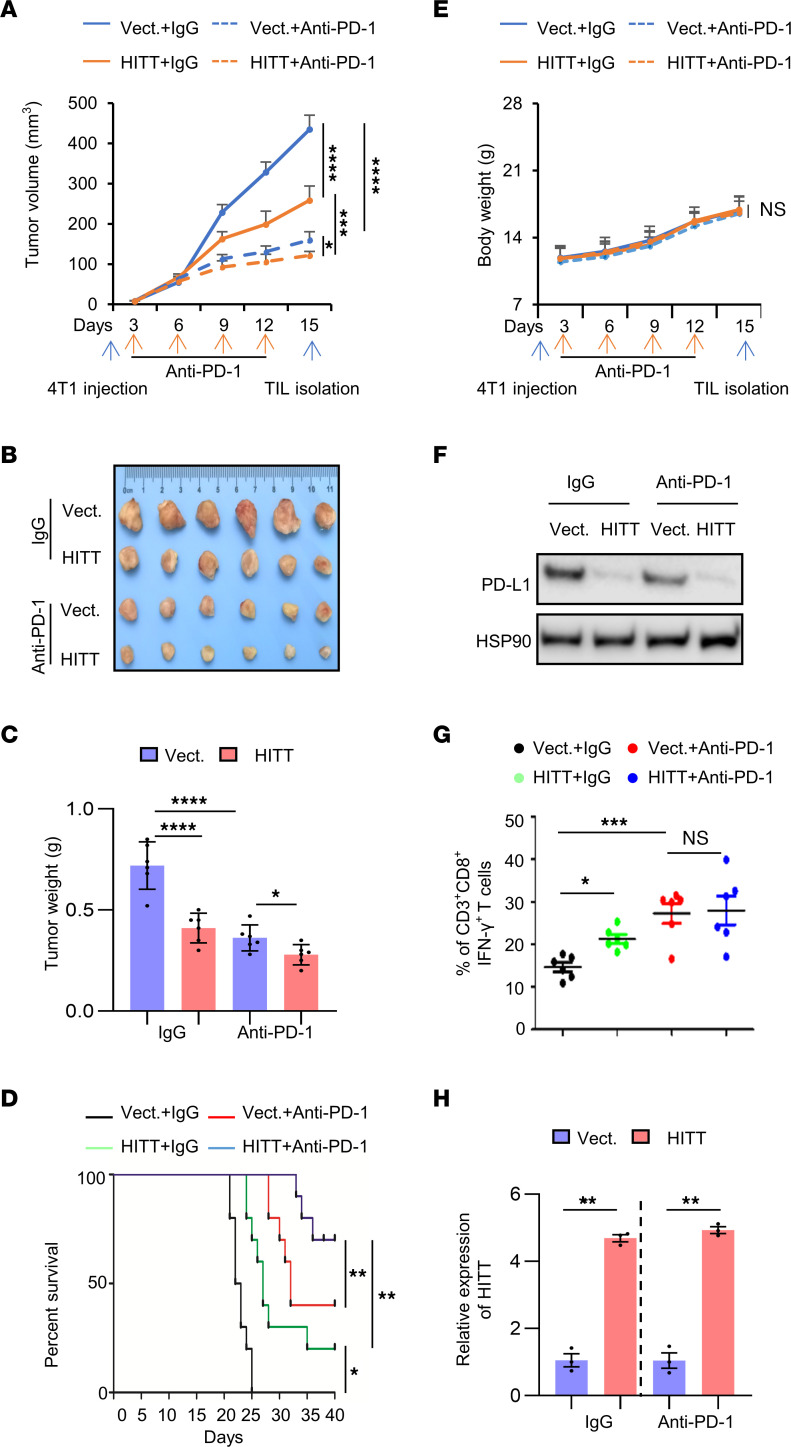
HITT inhibits tumor growth by attenuating PD-L1–mediated T cell deactivation in vivo. (**A**–**C**) Volume (**A**), images (**B**), and tumor weight (**C**). Each dot represents an evaluation in an individual tumor. (**D**) Kaplan-Meier survival curve of mice bearing syngeneic 4T1 tumor with treatment of IgG or anti–PD-1. (**E**) Body weights of BALB/c mice measured with treatments. (**F**) PD-L1 protein levels determined by WB. (**G**) Immunostaining of CD8^+^ IFN-γ^+^ in CD3^+^ T cell populations from isolated tumor-infiltrating lymphocytes in syngeneic tissues. Each dot represents an evaluation in an individual tumor. (**H**) HITT levels in 4T1 syngeneic determined by qRT-PCR. Data in **A**, **C**–**E**, and **G** are shown as mean ± SD. *P < 0.05; **P < 0.01; ***P < 0.001; ***P < 0.0001; NS, not significant by 2-way ANOVA (**A** and **E**, *n* = 6 mice per group), 1-way ANOVA (**C** and **G**, *n* = 6 mice per group), log-rank test (**D**, *n* = 10 mice per group), and Student’s *t* test (**H**). Data derived from 3 independent experiments are presented as mean ± SEM.

We next explored whether HITT promotes T cell immunity in vivo using the 4T1/immune-competent BALB/c orthotopic model of murine mammary carcinoma. HITT-overexpressing orthotopic tumors grew relatively slowly compared with control tumors (Figure 9, A–C). Anti–PD-1 antibody dramatically suppressed tumor growth compared with the corresponding controls. Intriguingly, the effect of HITT was compromised, but not completely abolished, by anti–PD-1 (Figure 9, A–C). The above data were validated using HITT-expressing lentivirus administration in PD-L1–KO tumors (Supplemental Figure 8, A–F). In contrast to HITT, PD-L1–5′-UTR binding defective HITT mutant (HITT-Mut) elicited little antitumor effect. Such a striking difference was completely abolished by PD-L1 KD (Supplemental Figure 8, D–F). HITT-overexpressing 4T1 tumor–bearing mice and anti–PD-1–treated mice survived significantly longer compared with control 4T1 tumor–bearing mice treated with IgG control (Figure 9D). Anti–PD-1–treated HITT-overexpressing 4T1 tumor–bearing mice survived longest among the 4 groups (Figure 9D). These data suggest that blocking PD-L1–mediated T cell inactivation by either anti–PD-1 antibody and/or HITT increases the survival of mammary tumor–bearing mice by suppressing tumor growth with low toxicity (Figure 9E).

Furthermore, HITT inhibited PD-L1 expression in orthotopic 4T1 tumors (Figure 9F and Supplemental Figure 8, G and H). In addition, a significant increase of the activated tumor-infiltrated CD8^+^ T cell population (CD3^+^CD8^+^IFN-γ^+^) was detected in HITT-overexpressing tumors (Figure 9G). Anti–PD-1 antibody had no obvious effects on HITT or PD-L1 expression (Figure 9H), while treatment led to a significant increase in the activated tumor-infiltrated CD8^+^ T cell population (Figure 9G). Anti–PD-1 antibody failed to further enhance the tumor-infiltrated CD8^+^ T cell population in HITT-overexpressing 4T1 tumors (Figure 9G). Unlike in the CD8^+^ T cell population, tumor growth and mouse survival were both further decreased or prolonged by the combination of anti–PD-1 and HITT overexpression (Figure 9, A–C).

### The association between HITT/RGS2 and PD-L1 in breast cancer tissues.

**Figure 10:**
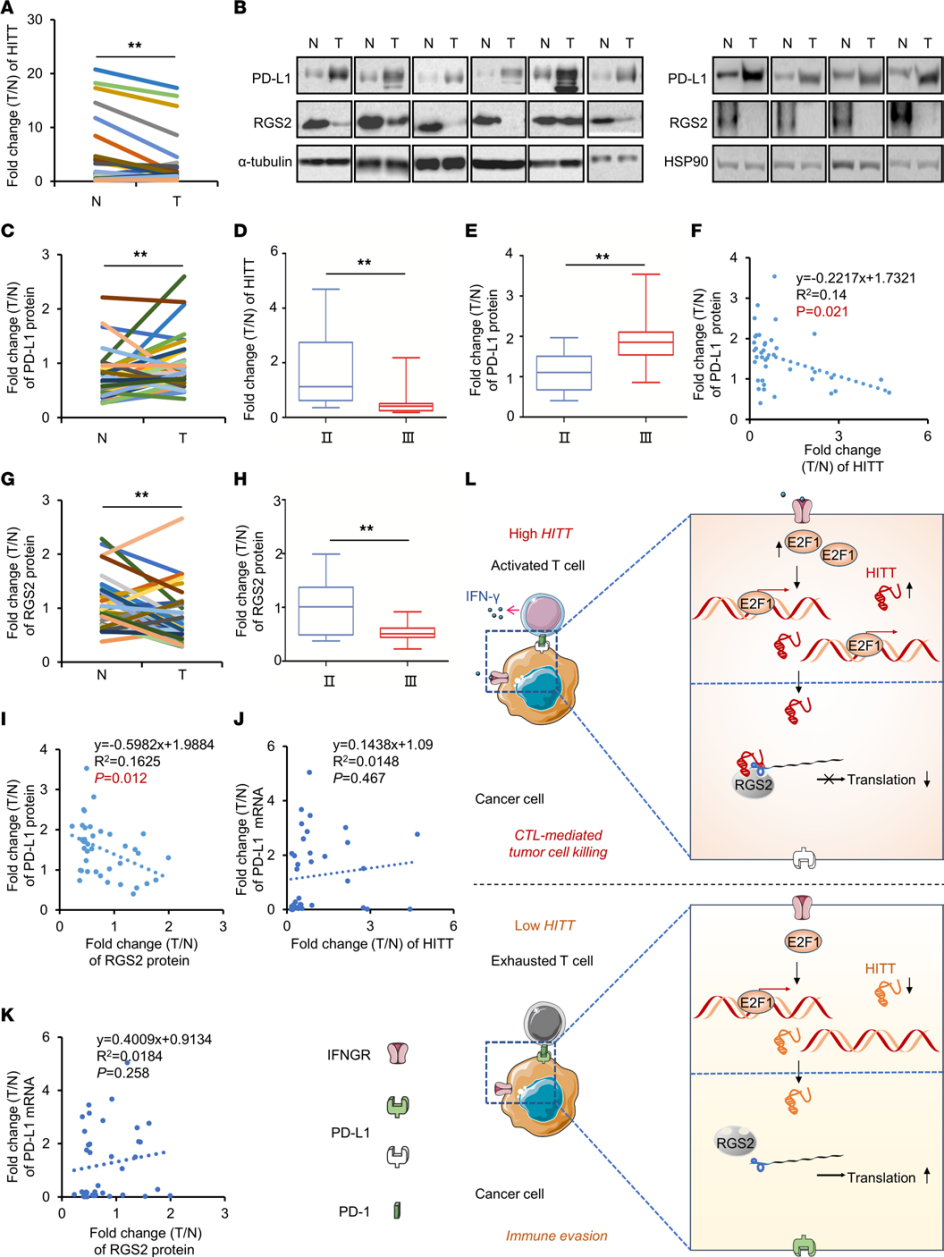
RGS2, HITT, and PD-L1 are associated with each other in vivo. (**A**) Expression of HITT in human breast tumors (T) and their paired adjacent normal controls (N) (*n* = 38) determined by qRT-PCR. (**B** and **C**) Representative WB (**B**) and quantification of PD-L1 proteins (**C**) in 38 pairs of breast cancer tissues and their adjacent normal controls. (**D** and **E**) The correlation between the fold change of HITT (**D**) and PD-L1 protein (**E**) and stages. (**F**) Lineal correlation analysis of the fold changes of HITT expression versus those of PD-L1 protein expression (*P* = 0.021). (**G**) Quantification of RGS2 proteins in 38 pairs of breast cancer tissues and their adjacent normal controls. (**H**) Correlation between fold change of RGS2 protein and TNM stages. (**I**) Lineal correlation analysis of fold changes of RGS2 protein expression versus those of PD-L1 protein expression (*P* = 0.012). (**J**) Lineal correlation analysis of fold changes of HITT expression versus those of PD-L1 mRNA expression. (**K**) Lineal correlation analysis of fold changes of RGS2 protein expression versus those of PD-L1 mRNA expression. (**L**) Schematic diagram of RGS2/HITT/PD-L1–regulated interaction between cancer cells and T cells to modulate tumor immunity. IFN-γ secreted by activated T cells or others triggers E2F1-mediated transactivation of lncRNA HITT in cancer cells, where HITT directly binds with RGS2 and PD-L1–5′-UTR. This function of HITT also strengthens the direct interaction between RGS2 and PD-L1–5′-UTR. These interactions among HITT, RGS2, and PD-L1–5′-UTR lead to a retarded translation of PD-L1 and elevated T cell activation. Such activity of HITT is impaired in cancer cells due to the reduced expression of HITT. Activating HITT in cancer cells is a potential treatment for elevating T cell immunity. Data derived from 3 independent experiments are presented as mean ± SEM (**A** and **C**–**K**). **P < 0.01, Student’s *t* test (**A**, **C**–**E**, **G**, and **H**). Correlations were calculated according to Pearson’s correlation (**F** and **I**–**K**).

qRT-PCR assay revealed that HITT was downregulated in breast cancer tissues compared with the adjacent normal controls (Figure 10A), while PD-L1 protein levels were increased in breast cancer tissues, as indicated by WB assays (Figure 10, B and C). The decreased HITT and increased PD-L1 were both associated with advanced stages of breast cancers (Figure 10, D and E). In addition, a negative association between the fold changes of HITT and those of PD-L1 protein was detected (Figure 10F). RGS2 was also found to be decreased in breast cancer tissues, and its downregulation was more evident in the advanced breast cancers (Figure 10, B, G, and H). Similarly to what occurred with HITT, RGS2 fold change exhibited a negative correlation with PD-L1 protein fold change (Figure 10I). Neither HITT nor RGS2 correlated with the mRNA levels of PD-L1 (Figure 10, J and K). Therefore, RGS2/HITT may contribute to PD-L1 regulation in vivo in human cancer tissues.

## Discussion

Here, we describe a mechanism that regulates PD-L1 translation: an IFN-γ–responsive lncRNA called HITT that, in coordination with RGS2, binds the *PD-L1–5*′-UTR, resulting in reduced mRNA translation, as indicated by the decreased occupancy of *PD-L1* mRNA by polysomes and reduced de novo protein synthesis. In addition, arbitrarily increasing HITT expression in cancer cells promotes T cell–mediated cancer-killing effects by inhibiting the PD-1/PD-L1 axis both in vitro and in vivo. Furthermore, a negative association between HITT/RGS2 and PD-L1 expression was detected in vivo in human breast cancers, suggesting that HITT may inhibit PD-L1 expression in vivo (Figure 10L). Thus, translational suppression of PD-L1 expression by HITT/RGS2 may represent an alternative strategy against cancer and a marker for prediction of the anti–PD-1/PD-L1 response.

Previous studies have indicated that constitutive expression of PD-L1 on cancer cells, despite it having a defined role in tumorigenesis, is less reliable than inflammation-induced PD-L1 expression for the prediction of response to immunotherapy (25). In terms of anti–PD-1/PD-L1 therapies, it is essential that we understand the regulatory mechanism behind IFN-γ–increased PD-L1 expression. Interestingly, HITT is activated by IFN-γ in the microenvironment. Although inflammation simultaneously elevates PD-L1 and HITT expression, HITT markedly relieves PD-L1 elevation induced by IFN-γ. These data suggest that IFN-γ–induced pro- and antiimmunity factors are interconnected and regulate overall functional output of IFN-γ. Moreover, HITT restrains PD-L1 expression in a variety of cancer types, suggesting that HITT’s inhibition of PD-L1 expression is a broad mechanism. Considering the ability of HITT to respond to IFN-γ signals and the improved response of HITT-overexpressing cancer cells or tumors to anti–PD-1 treatment, it is worth investigating whether HITT can predict response to anti–PD-1/PD-L1 treatment in future studies. In addition, HITT is sensitive to diverse cancer-related stimuli and its activity is regulated by several different mechanisms (22, 23). Here, we found that E2F1, but not EGR1, is required for the transcriptional activation of HITT upon IFN-γ stimulation. This finding is consistent with the notion that E2F1 is a transcription factor that is important in the inflammatory response (29). Whether or not EGR1 activation upon other inflammatory signals contributes to the activation of HITT and subsequent immune surveillance needs to be investigated in the future.

Notably, although HITT overexpression and an anti–PD-1 monoclonal antibody have similar effects on T cell activity, their combination leads to a synergetic effect that inhibits tumor growth and prolongs the survival of mice bearing 4T1 tumors. Given the remarkable effect of HITT on T cell activity and the synergetic effect observed in combination with anti–PD-1 antibody therapy, it would be worth evaluating the therapeutic potential of the lncRNA HITT.

In addition, although mechanisms of PD-L1 regulation have not been fully investigated, recent studies suggest that cancer cells utilize comprehensive mechanisms to fine-tune PD-L1 expression. For example*,* STAT3, C-Myc, HIF-1α, c-JUN, and NF-κB increase PD-L1 expression at the transcriptional level. CSN5, GSK3β, CDK4/CDK6, CMTM4/6, and B3GNT have been shown to regulate PD-L1 degradation (30). Connection between PD-L1 expression and lncRNAs has also been suggested. Some lncRNAs were found to regulate PD-L1 mRNA levels by targeting microRNAs. Recently, Mineo et al. reported that lncRNA *INCR1* is activated in response to IFN-γ and promotes PD-L1 transcription in cis by binding with *HNRNPH1* (31). Another lncRNA, *lncMX1–215*, is induced by IFN-γ and regulates PD-L1 transcription via an epigenetic mechanism (32). For what we believe is the first time, a lncRNA (HITT) has been shown to directly connect with PD-L1 translation. In support of our data, Suresh et al. and Xu et al. have demonstrated the essential contribution of *PD-L1* mRNA translation in controlling its expression (33, 34). Of note, although alterations in translation normally lead to mRNA degradation (35), there are a few exceptions. HITT inhibits PD-L1 translation, while having no obvious impacts on its mRNA levels, which provides another example of the independent regulation of translation and mRNA stabilization. These data, together with our findings in this study, are coherent with the emerging idea that translation is an efficient mechanism that dynamically controls protein abundance with the advantage of promoting a response.

Mechanistically, our results demonstrate that HITT’s reduction of PD-L1 translation relies on the inhibition of cap-dependent initiation. However, BS2-mediated HITT/PD-L1–5′-UTR interaction is required but not sufficient for the optimal inhibition of PD-L1. Based on the features of HITT in activating T cell immunity and in inhibiting PD-L1 translation, proteins possibly involved in this process were screened in the GO database, which was followed by a literature search. Interestingly, among such proteins, RGS2 is uniquely required for HITT-inhibited PD-L1 translation. Notably, RGS2 is reported to bind with eIF2Bε to fulfil its role in regulating mRNA translation, yet RGS2 inhibits PD-L1 expression in eIF2Bε KD cells, which implies that RGS2 has a novel translation regulatory mechanism (16). Indeed, for what we believe is the first time, we report an RNA-binding activity of RGS2, which is required for inhibition of PD-L1 translation. HITT/RGS2 regulates PD-L1 translation in a *PD-L1–5*′-UTR-dependent manner. HITT, RGS2, and *PD-L1–5*′-UTR interact with each other. HITT and RGS2 are interdependent in regulating PD-L1–5′-UTR reporter activity and PD-L1 translation. Based on these results, we propose a model showing that pairwise interaction of HITT/RGS2/*PD-L1–5*′-UTR is essential for impairing PD-L1 translation under both basal and IFN-γ–stimulated conditions. This model was further validated by examining PD-L1 expression or *PD-L1–5*′-UTR luciferase activity using binding-defective RGS2, HITT, or *PD-L1–5*′-UTR mutants, as shown in Figure 7. The multiple factors involved in regulation allow precise and selective control of PD-L1 expression. It should also be noted that lncRNA is normally very low abundance. Thus, the question arising from the data presented is how to reconcile the low abundance of HITT with its apparent functional importance by interacting with PD-L1 mRNA. Whether HITT is concentrated by phase separation warrants further investigation. In addition, HITT may initiate the inhibitory reaction on PD-L1 expression. This may be followed by translational inhibition mediated by additional unknown factors, which may amplify the inhibitory signal to PD-L1 translation even when HITT is released from the PD-L1–5′-UTR complex. This model is also worthy of further exploration.

In support of a role for RGS2 in regulating T cell immunity, a previous report has shown that *rgs2*^−/−^ mice have abnormal T cell immunity, which the authors propose may be due to increased cAMP levels in T cells mediated by loss of RGS2 (17). To date, RGS2 has only been implicated in the regulation of T cell activity. In our study, we demonstrate the activity and mechanism by which RGS2 expression in cancer cells regulates immune surveillance.

Moreover, in agreement with the finding that increased PD-L1 expression is associated with poor outcomes in breast cancer patients, our data also reveal the predictive value of PD-L1. Oncogene signals, such as Myc overexpression, Ras activation, loss of PTEN, or PI3K/Akt mutation, contribute to the constitutive activation of PD-L1 in cancer cells (30). Our data provide an alternative explanation for PD-L1 dysregulation, because the decreased expression of HITT is inversely correlated with PD-L1 expression in breast cancer tissues, and the inhibitory activity of HITT on PD-L1 expression can be demonstrated both in vitro and in orthotopic models.

Together, our data elucidate a distinctive mechanism by which PD-L1 expression is regulated and uncover antitumor activity of HITT and RGS2 through the prevention of tumor cell immune escape. Our research provides insight into the network that regulates immunosuppression and may enhance the antitumor effects of immune checkpoint blockade therapies.

## Methods

### Human breast cancer tissues.

Human breast cancer tissues and their corresponding adjacent normal controls were collected from Qilu Hospital of Shandong University. Specimens were collected and stored in liquid nitrogen immediately after surgery.

### Animal experiments.

BALB/c mice (6 week-old females) were purchased from Beijing HFK Bioscience Co. Mice were randomly divided into 4 groups, and 50,000 4T1 cells in 100 μL 1× PBS were injected into mammary fat pads. To block PD-L1/PD-1 signaling, 100 μg anti–PD-1 antibody was injected intraperitoneally into mice at 3, 6, and 9 days after tumor inoculation, with IgG as a negative control (36). To block CD8^+^ T cell function, 3 days after tumor inoculation, 20 μg monoclonal anti-CD8α antibody was administered via intraperitoneal injection every other day for 3 weeks (37). For the HITT-expressing lentivirus antitumor treatments, mice bearing similar sizes of tumor (80 mm^3^) were randomly divided into 5 groups: (a) PBS, (b) lenti-Vect+IgG, (c) lenti-HITT+IgG, (d) lenti-Vect+anti–PD-1 antibody, and (e) lenti-HITT+anti–PD-1 antibody. PBS alone, lentiviruses (1 × 10^8^ PFU), and IgG or anti–PD-1 antibody (100 μg) in 100 μl 1× PBS were administered intratumorally at 3 sites per tumor. The treatments were repeated 4 times every 2 days. Tumor volume was measured every 3 days with a caliper using the following formula: π/6 × length × width^2^ (38). At the end point, the tumor was carefully peeled, photographed, and weighed. Protein, RNA, and T cells were collected for further analysis.

### Cell culture, stable transfectants, and transfection.

Human breast cancer (MDA-231, MDA-453, MDA-468, BT549, BT474, MCF7, T47D), colorectal cancer (HT29), cervical cancer (HeLa), lung cancer (H23, H1299), and mouse breast cancer (4T1) cells were purchased from ATCC and cultured in RPMI-1640 medium (Gibco, Thermo Fisher Scientific) or DMEM supplemented with 10% (v/v) FBS (Biological Industries). All cells were cultured in the humidified incubator at 37°C under 5% CO_2_. Stable cell lines overexpressing HITT and the vector control were established as previously described. For the transient transfection, the indicated plasmid constructs or siRNAs were introduced into cells with Lipofectamine 2000 (Life Technologies) according to the manufacturer’s instructions. At 48 to 72 hours after transfection, cells were subjected to the indicated treatments or analyses. For IFN-γ treatment, cells were serum starved overnight prior to stimulation at the indicated time periods and concentrations. Plasmids used in this study are listed in Supplemental Table 4.

### Lentivirus production.

HITT was inserted into the lentivirus vector pLnc-KP. The 3,000 ng pLnc-KP control or recombined pLnc-KP-HITT were transfected into 293T cells with 1,500 ng pGag/pol, 900 ng pVSVG, and 600 ng pRev lentiviral packing vectors, respectively, using Lipofectamine 2000 according to the manufacturer’s instructions. Forty-eight hours after transfection, supernatant was collected and centrifuged at 4000*g* for 10 minutes and then filtrated with a 0.45 nm filter to harvest the lentivirus particles.

### T cell–mediated tumor cell-killing assay.

The assay was performed according to previous reports (25, 39). Briefly, human PBMCs obtained from 3 different healthy donors from Harbin Blood Institute were maintained in F12-K medium supplemented with 10% FBS. T cells were activated by treating PBMCs with anti-CD3 antibody (100 ng/ml), anti-CD28 antibody (100 ng/ml), and IL-2 (10 ng/ml) for 48 hours (40, 41), and 5 × 10^5^ cancer cells were seeded in a 24-well plate. Twenty-four hours later, 5 × 10^6^ activated T cells (10:1) were seeded and cocultured with the indicated cancer cells for an additional 6 hours. Then cells were washed twice with 1× PBS to discard T cells and suspend dead cancer cells. The remaining living cells were fixed with 4% formaldehyde for 30 minutes at room temperature and stained with 0.1% crystal violet solution for 20 minutes. After 4 washes with 1× PBS, the plates were photographed and quantified. Alternatively, T cell cytotoxicity activity was determined using the MTS Reagent Kit following the manufacturer’s instructions (CellTiter 96 AQueous One Solution Cell, Promega).

OT-I T cell–based tumor-killing assays were performed as described previously (25). C57BL/6-Tg (TcraTcrb) 1100Mjb/J (OT-I) mice were purchased from Shanghai Model Organisms Center Inc. The mice express a T cell receptor recognizing an H-2b–restricted OVA 257–264 epitope, SIINFEKL. For OT-I T cell isolation, the spleen was homogenized and the single splenocytes were pelleted and suspended in red blood cell lysis buffer (0.15 M NH_4_CL, 10 mM KHCO_3_, 0.1 mM Na_2_EDTA). Then splenocytes were resuspended at a density of 2 × 10^6^/ml in RPMI culture medium containing 1 μg/ml OVA 257–264 peptide, 5 μg/ml mouse recombinant IL-2, and 40 μM 2-mercaptoethanol. OT-I T cells were isolated and purified by mouse CD8^+^ T cell MicroBeads (Miltenyi Biotec) after incubation at 37°C for 5 days. The FACS assay confirmed that over 90% were CD8^+^ T cells. OVA-expressing 4T1 cells were established by introducing OVA into 4T1 cells (4T1-OVA), which were seeded overnight. OT-I T cells were added into the culture (4T1-OVA: OT-I T, 1:4). The OT-I T cell–mediated 4T1-OVA cell-killing effect was evaluated by crystal violet staining 48 hours after the addition of T cells. Images were quantified using ImageJ (NIH, version 1.52a).

### ELISA of IL-2 and IFN-γ.

20,000 Cancer cells were seeded in 96-well plates. The cancer cells and T cells were washed with 1× PBS to eradicate contaminating traces of IFN-γ or IL-2 in the culture medium. 10,000 Activated T cells were incubated with the cancer cells in 96-well plates for an additional 72 hours, and 10 μg/ml of anti–PD-1 antibody or IgG control was added in the coculture system where indicated. 100 μl or 200 μl total supernatant was subjected to the measure of the secreted IL-2 and IFN-γ protein using IL-2/IFN-γ kits (Human Quantikine IL-2/IFN-γ ELISA Kits, R&D Systems) according to the manufacture’s instructions. Each experiment was repeated 3 times.

### qRT-PCR assay.

Cells were washed twice with 1× PBS, and then total RNA was extracted using TRIzol Reagent (Takara); 2 μg purified RNA was used to synthesize cDNA according to the manufacturer’s protocol (Prime Script RT Reagent Kit with gDNA Eraser). qPCR was performed in triplicate with the ViiA7 Real-Time PCR instrument (Applied Biosystems) using the SYBR Premix Ex Taq II Kit (RR820L; Takara). Relative expression levels of the targeted genes compared with *18S* rRNA or *GAPDH* were calculated using the 2^−ΔΔCT^ method. The primer sequences used for RT-PCR are listed in Supplemental Table 5.

### WB assay.

Cells or tissue samples were lysed with UREA buffer (8M urea, 1M thiourea, 0.5% CHAPS, 50 mM DTT, and 24 mM spermine) and fully vibrated for 30 minutes at room temperature. The same amounts of proteins were separated by SDS-PAGE). After transferring, PVDF membrane with proteins was incubated with the indicated primary antibodies and secondary antibodies, protein signals were visualized by ECL (32106, Thermo Scientific), and images were captured by the Image Studio System (ECL, LI-COR). Antibodies are listed in Supplemental Table 6.

### Luciferase reporter assay.

Luciferase reporter gene expression plasmids and the Renilla-luciferase control plasmid were transfected into cells. Forty-eight hours after transfection, cells were harvested using luciferase lysis buffer and subjected to analysis with the Dual Luciferase Reporter Assay according to the manufacturer’s protocols (Promega, E1910). Luciferase reporter activities were determined as the ratio of the target gene luciferase to the renilla-luciferase control.

### ChIP.

Briefly, cells were pretreated with 1% formaldehyde in the culture media for 20 minutes at 37°C to yield protein-DNA crosslink complexes, and then the complexes were extracted and sonicated in the ChIP lysis buffer. Purified chromatin was equally separated and incubated with either anti-E2F1 antibody or IgG control overnight at 4°C. Thereafter, the immunoprecipitates were collected by centrifugation at 800*g* and the resulting protein-DNA complexes were decrosslinked at 65°C. After 4 washes in 1× PBS, the fragmented DNA was extracted using the Axygen Product Purification Kit and subjected to PCR analysis.

### AHA labeling to identify newly synthesized proteins.

MDA-231 cells were washed 3 times in 1× PBS and then incubated in methionine-free medium for 30 minutes to wipe off residual methionine. Then cells were incubated with 50 μM AHA (Invitrogen) at 37°C for 4 hours. After the treatments, cells were sonicated followed by centrifugation at 13,000*g* for 30 minutes, and 50 mg of the resulting supernatant was subjected to the treatment with click reactions (Click-iT Protein Reaction Buffer Kit; Invitrogen). Total proteins from click reactions were pelleted by centrifugation at 800*g* for 5 minutes in the presence of methanol/chloroform, and the resolubilized proteins were incubated with 50 μl of streptavidin-coupled magnetic beads for 5 hours at room temperature. Proteins linked with magnetic beads were boiled in 30 μl 5× loading buffer for 10 minutes at 100°C and then subjected to WB analysis.

### Polysome profiling.

3×10^7^ Cells were treated with 0.1 mg/ml cycloheximide (CHX) for 5 minutes, before lysing in polysome lysis buffer (15 mM Tris-HCL PH 7.5, 15 mM MgCl_2_, 0.3M NaCl, 1% Triton X-100, 0.1 U/μl RNA inhibitor, 100 μg/ml CHX, 1 μg/ml heparin, and 1× protease inhibitor cocktail). Nuclei and membrane debris were removed by centrifugation at 10,000*g* for 5 minutes, and lysate was loaded across sucrose gradients. The sucrose gradient samples were obtained by centrifugation at 192,000*g* for 2 hours at 4°C using SW40Ti rotor in the Beckman Optima XPN Ultracentrifuge, and fractionated RNA samples were monitored by using an ultraviolet spectrophotometer at 254 nm. RNA in each sucrose gradient was collected and extracted in 3 volumes of TRIzol, followed by qRT-PCR assay for the indicated genes.

### CLIP.

Cells were washed twice in 1× PBS and then subjected to UV crosslinking at 400 mJ/cm^2^. The UV crosslinked cells were lysed in the lysis buffer (50 mM Tris-HCl [pH 8.1], 85 mM KCl, 10 mM EDTA, 5 mM PIPES [pH 8.0], 1% SDS, and 0.5% NP40) supplemented with Protease Inhibitor Cocktail and RNase inhibitor (Thermo Fisher). Total lysates were precleaned by protein G sepharose beads at 4°C for 1 hour. The supernatant was collected and incubated with the indicated primary antibodies or IgG control, rotating at 4°C overnight. The next day, the antibody-RNA complexes were collected and incubated with the blocked protein A/G sepharose beads for 1 hour. After that, the immunoprecipitated RNA was eluted, isolated, and reverse transcribed to cDNA for the subsequent qRT-PCR analysis.

### In vitro RNA pull-down assay.

Biotin-labeled RNA was synthesized in vitro using Biotin RNA Labeling Mix (Roche, 11685597910). After treatment with RNase-free DNase I, biotin-labeled RNA was heated at 95°C for 2 minutes followed by 3 minutes of incubation on ice to recover the secondary structure of RNA. The RNA was then incubated with streptavidin agarose beads (Invitrogen) overnight. The fresh cell lysates were collected and added to RNA-captured beads, and the mixture was incubated at 4°C for 1 hour. After 4 washes in 1× PBS, the beads were boiled at 95°C for 5 minutes in SDS loading buffer and the associated proteins were detected by WB assay.

### PD-1/PD-L1 interaction assay.

Briefly, 72 hours after HITT KD, MDA-231 cells were washed twice in 1× PBS and fixed with 4% paraformaldehyde for 20 minutes at room temperature. Cells were incubated with 5 μg/ml recombinant human PD-1 Fc protein at 4°C overnight, followed by additional incubation with the anti-human Alexa Fluor 488 dye–conjugated secondary antibody for 30 minutes at room temperature. Then nuclei were stained with DAPI at room temperature for 5 minutes. After incubation with PD-1 Fc protein, the following process was protected from exposure to light. Images were acquired by a Zeiss confocal microscope (LSM880) and afterwards counterstained with DAPI at room temperature for 5 minutes.

### PLA.

Cells grown on coverslips were permeabilized with 1% saponin (w/v) for 1 hour at room temperature, followed by blocking with blocking buffer (10 mM Tris-acetate, pH 7.5, 10 mM magnesium acetate, 50 mM potassium acetate, 250 mM NaCl, 0.25 μg/μL BSA, and 0.05% Tween 20) in the presence of 20 μg/mL sheared salmon sperm DNA (sssDNA) at 4°C for 1 hour; 100 nM specific RNA probes were added to fresh blocking buffer, heated at 70°C for 3 minutes, and incubated with fixed/permeabilized cells at 37°C for 1 hour. Subsequently, the cells were blocked in 1× PBS with 0.1% Tween 20 containing 1% (v/v) BSA and 20 μg/mL sssDNA at room temperature for 1 hour. After that, cells were incubated with anti-RGS2 and anti-biotin antibodies derived from different species at 4°C overnight at a dilution rate of 1:50. The subsequent PLA ligation and amplification steps were performed according to the manufacturer’s instructions (Duolink In Situ PLA Kit; Duo92004, Duo92002, Duo92008; MilliporeSigma). The probe sequences used in PLA are listed in Supplemental Table 7.

### FISH.

FISH was performed following the manufacturer’s instructions (Gene Pharma). Briefly, after IFN-γ stimulation, HeLa cells were fixed in 4% PFA solution at room temperature for 15 minutes. The cells were treated with 0.1% buffer A (0.1% Triton X-100) at room temperature for 15 minutes followed by another round of incubation in buffer C (2 × SSC) at 37°C for 30 minutes. Then slides were incubated with denaturated FAM-labeled PD-L1–5′-UTR and Cy3-labeled-HITT probes (8 μM final concentration) in buffer E (1× SSC, 35% formamide, 10% dextran sulfate) at 37°C overnight and then washed sequentially with buffer F (0.1% Tween 20) and buffer C at 42°C for 5 minutes each. Finally, images were acquired by a Zeiss confocal microscope (LSM880) after being counterstained with DAPI at room temperature for 5 minutes. The probe sequences used in FISH assays are listed in Supplemental Table 7.

### Tumor-infiltration lymphocyte analysis.

Tumor-infiltration lymphocyte profile analysis was conducted following the protocol described previously (25). Briefly, 4T1 syngeneic tumors dissected from mice were digested in collagenase/hyalurinidase (STEMCELL Technologies) and DNase (MilliporeSigma), and T cells were enriched sequentially on a Ficoll gradient (MilliporeSigma) using a Dynabeads Untouched Mouse T Cell Kit (Invitrogen). The isolated T cells were fixed with 4% paraformaldehyde for 5 minutes and stained with PE-CD3ε (145-2C11; BioLegend), PE–cyanine7–IFN-γ (XMG1.2; BioLegend), and FITC-CD8a (53-6.7; BD Biosciences — Pharmingen) for 30 minutes at room temperature. After being washed 3 times, the populations of infiltrated T cells were detected and analyzed with a BD FACS (LSRF Fottessa) cytometer.

### Data availability.

Mass-spectrum data were deposited in the ProteomeXchange Consortium via the iProX partner repository (PXD039107).

### Statistics.

Data are represented as mean ± SEM or SD. Statistical significance of differences between 2 groups was evaluated by 2-tailed Student’s *t* test, while statistical significance of differences among multiple groups was analyzed by ANOVA using GraphPad Prism software, version 8.0.2. Correlations were calculated according to Pearson’s statistical analysis. Significance of survival difference was determined by the log-rank test (*n* = 10 per group). *P* values of less than 0.05 were considered statistically significant.

### Study approval.

The experiments with BALB/c mice were conducted according to protocols approved by the Rules for Animal Experiments published by the Chinese Government and approved by the Research Ethics Committee of Harbin Institute of Technology. Written, informed consent was obtained from all patients. The study was approved by the Research Ethics Committee of Shandong University.

## Author contributions

YH designed and supervised the project and wrote the paper. Q Lin, XW, GH, ZX, WZ, DZ, ML, and TG performed the experiments. Q Lin, GH, ZX, SZ, HL, and DZ analyzed the data. Q Lin and Q Leng performed animal experiments. TL and XZ collected clinical breast cancer samples and analyzed clinical data.

## Supplementary Material

Supplemental data

## Version 1 04/04/2023

In-Press Preview

## Version 2 06/01/2023

Electronic publication
