# Supplementary material for: Long noncoding RNA HITT coordinates with RGS2 to inhibit PD-L1 translation in T cell immunity
Source: J Clin Invest. 2023 Jun 1;133(11):e162951. doi: 10.1172/JCI162951 (PMC10231998; doi:10.1172/JCI162951)
Supplement: Supplemental data [file jci-133-162951-s087.pdf]

Long noncoding RNA HITT coordinates with RGS2 to inhibit PD-L1 translation in T cell immunity

Qingyu Lin[1*], Tong Liu[2*], Xingwen Wang[1], Guixue Hou[3], Zhiyuan Xiang[1], Wenxin Zhang[1], Shanliang Zheng[1], Dong Zhao[1], Qibin Leng[4], Xiaoshi Zhang[5], Minqiao Lu[1], Tianqi Guan[1], Hao Liu[1], Ying Hu [1#]

1 School of Life Science and Technology, Harbin Institute of Technology, Harbin, Heilongjiang Province, China, 150001

2 Department of Breast Surgery, Harbin Medical University Cancer Hospital, Harbin, China; Heilongjiang Academy of Medical Sciences, Harbin, China.

3 BGI-SHENZHEN, Shenzhen, 518083, China.

4 Affiliated Cancer Hospital and Institute of Guangzhou Medical University, State Key Laboratory of Respiratory Disease, 78 Heng Zhi Gang Road, Guangzhou 510095, China.

5 Department of Clinical Laboratory, Qilu Hospital of Shandong University, Jinan, Shandong, China 250012

Conflict of interest: The authors have declared that no conflict of interest exists.

* These authors contribute equally to the work.

Address correspondence to: School of Life Science and Technology, Harbin Institute of Technology, 150001 Harbin, Heilongjiang Province, China. Tel: 0086-86403826. Email: huying@hit.edu.cn.

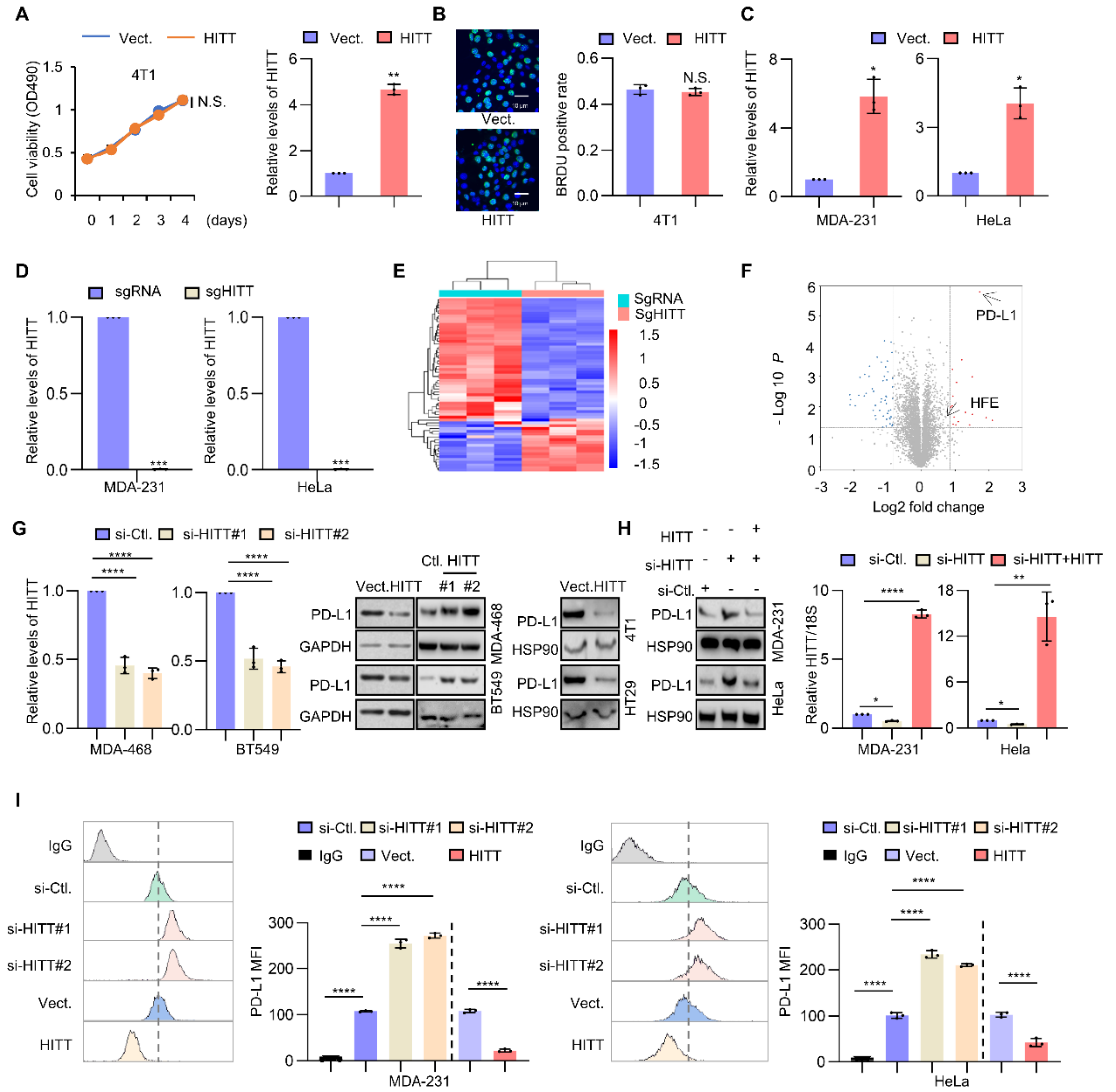

Supplemental Figure 1 HITT inhibits PD-L1 expression.

(**A** and **B**) The viability and proliferation rate of 4T1 cells were measured by MTT (**A**) and BrdU incorporation assays (**B**) after stable transfection of HITT or vector control. (**C** and **D**) HITT overexpression (**C**) and knockout (KO) (**D**) efficiencies were confirmed by qRT-PCR in MDA-231 and HeLa cells. (**E**) A clustering heatmap illustrating the differentially expressed protein classifications of SgRNA and SgHITT samples. The different color represents protein different expression levels of SgHITT group compared to the SgRNA group. (**F**) Volcano plot for the comparison differentially expressed proteins between the SgRNA group and SgHITT group. The cutoff values fold change >1.8 and *P* value < 0.05 were utilized to identify differentially expressed proteins. Non-changed proteins were shown in grey color. Red color is indicative of up-regulated proteins and blue is indicative of

down-regulated proteins. (**G**) PD-L1 levels were measured by western blot assay in MDA-468, BT549, HT29 and 4T1 cells after overexpression or KD HITT. (**H**) PD-L1 protein levels were analyzed by western blot (left) and HITT KD and recovery efficiency were confirmed by qRT-PCR (right). (**I**) PD-L1 levels were determined by measuring membrane PD-L1 through flow cytometry. Data are derived from three independent experiments shown as mean ± SEM. *P < 0.05; **P < 0.01; ***P < 0.001; ****P < 0.0001; N.S. not significant by two-way ANOVA test (**A**) and Student's t test (**A**-**D**) and one-way ANOVA test (**G**-**I**).

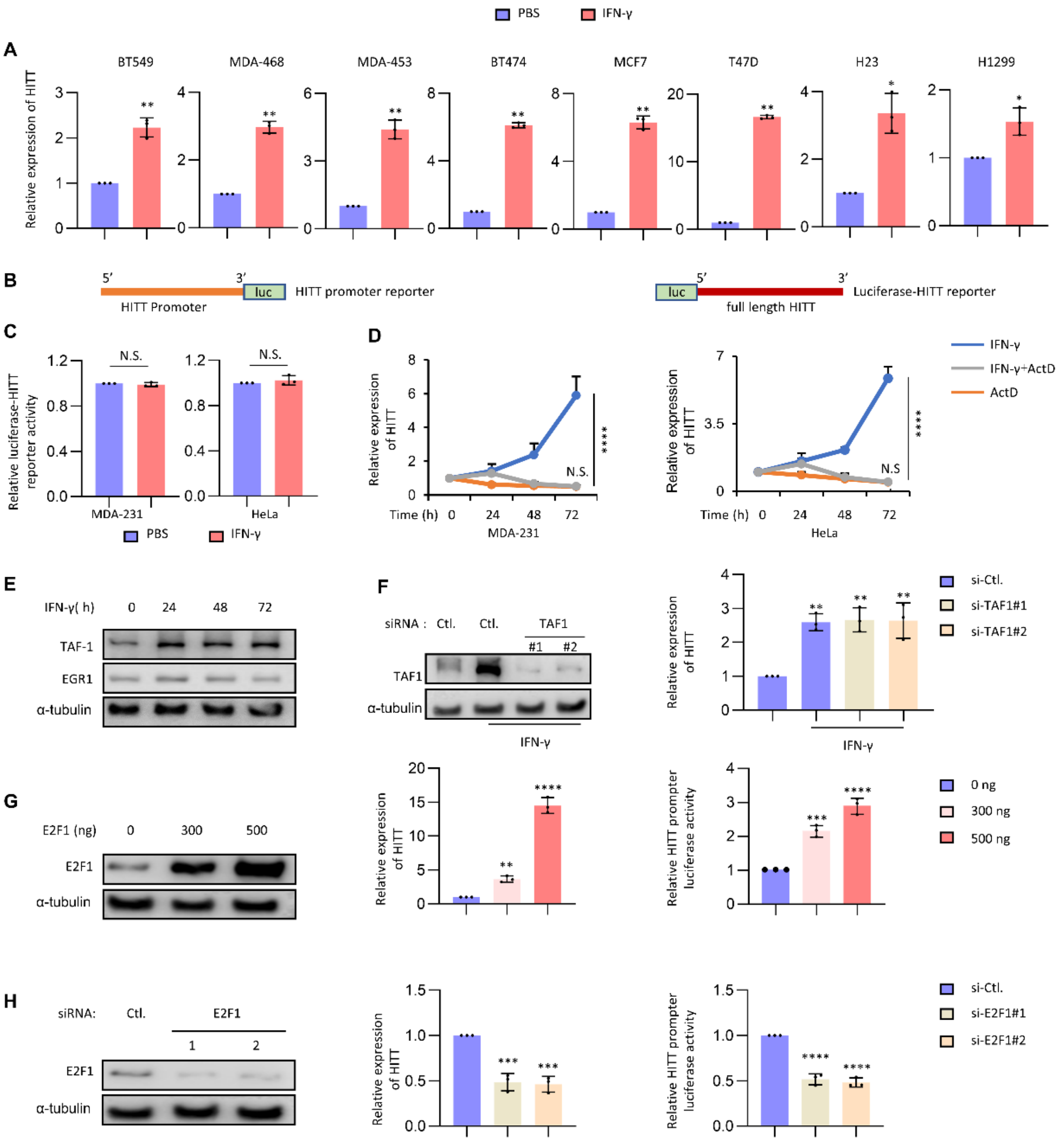

Supplemental Figure 2 HITT is induced by E2F1 under the IFN-γ treatment

(**A**) HITT levels were measured by qRT-PCR in different cancer cell lines under the treatment of 10ng/ml IFN-γ for 24h. (**B**) Schematic of HITT promoter-driven luciferase reporter (HITT promoter reporter) and luciferase-full length HITT reporter (luciferase-HITT reporter) constructs. (**C**) Luciferase-HITT reporter activities were measured by luciferase reporter assay in MDA-231 and HeLa cells under the treatment of 10ng/ml IFN-γ for 24h. (**D**) HITT levels were measured in MDA-231 and HeLa cells by qRT-PCR after treatment of different time periods of IFN-γ (10ng/ml) in the presence or absence of RNA synthesis inhibitor Actinomycin D (ActD). (**E**) The protein levels of TAF1 and EGR1 were determined by western blot following the treatment of IFN-γ (10ng/ml) in HeLa cells for the different time periods. (**F**) HITT levels were measured by qRT-PCR in TAF1 knockdown

(KD) HeLa cells in the presence of IFN-γ (10ng/ml for 24h). (**G** and **H**) HITT levels and HITT promoter luciferase activities were measured by qRT-PCR and luciferase reporter assay, respectively, following introducing ascending levels of E2F1 by transfection with 0, 300 or 500ng expression plasmid of E2F1 (**G**) or E2F1 KD (**H**) in HeLa cells. The overexpression (left, **G**) or KD (left, **H**) efficiency of E2F1 was determined by western blot assay. Data are derived from three independent experiments shown as mean ± SEM. *$P < 0.05$; **$P < 0.01$; ***$P < 0.001$; ****$P < 0.0001$; N.S. not significant by Student's t test (**A**, **C**) and two-way ANOVA test (**D**) and one-way ANOVA test (**F**-**H**).

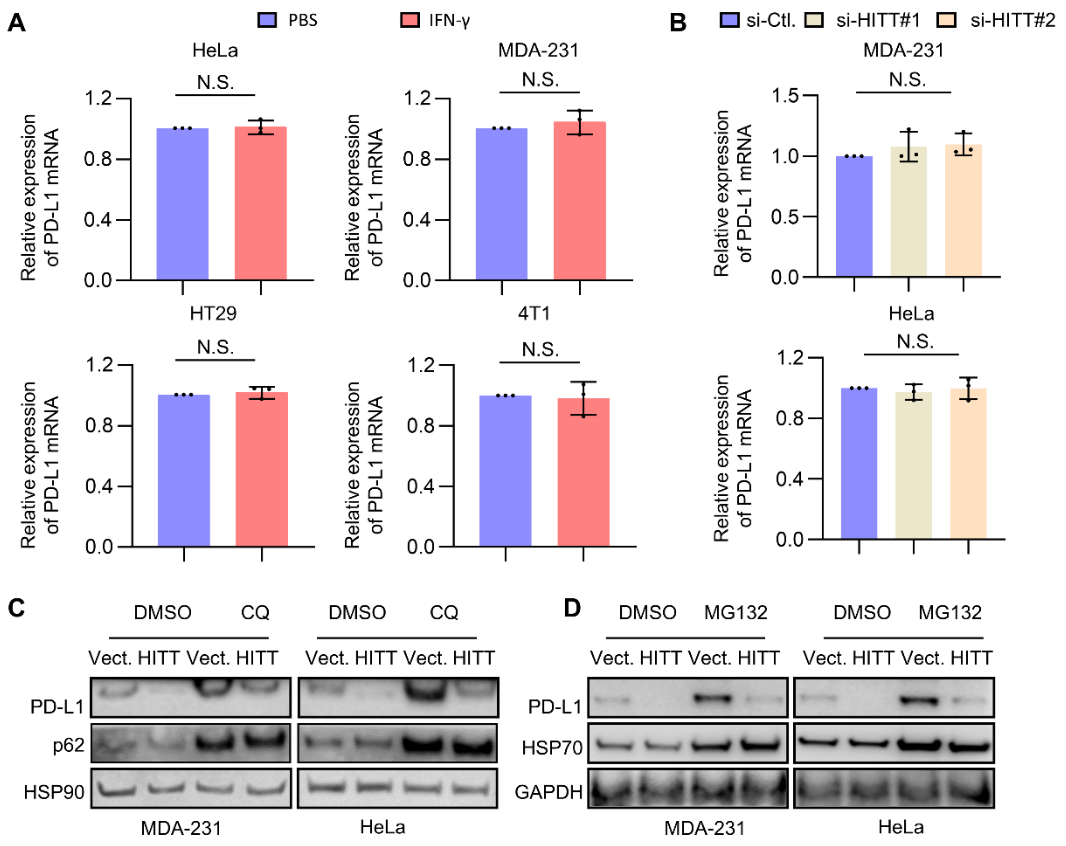

Supplemental Figure 3 HITT limits PD-L1 translation

(**A** and **B**) PD-L1 mRNA levels were measured by qRT-PCR in HITT stable lines (**A**) or HITT KD (**B**) cells as indicated. (**C** and **D**) PD-L1 levels were measured by western blot in HITT stable lines or HITT KD cells in the presence or absence of Chloroquine (CQ) (**C**) or MG132 (**D**). Data are derived from three independent experiments shown as mean ± SEM. N.S. not significant by Student's t test (**A**) and one-way ANOVA test (**B**).

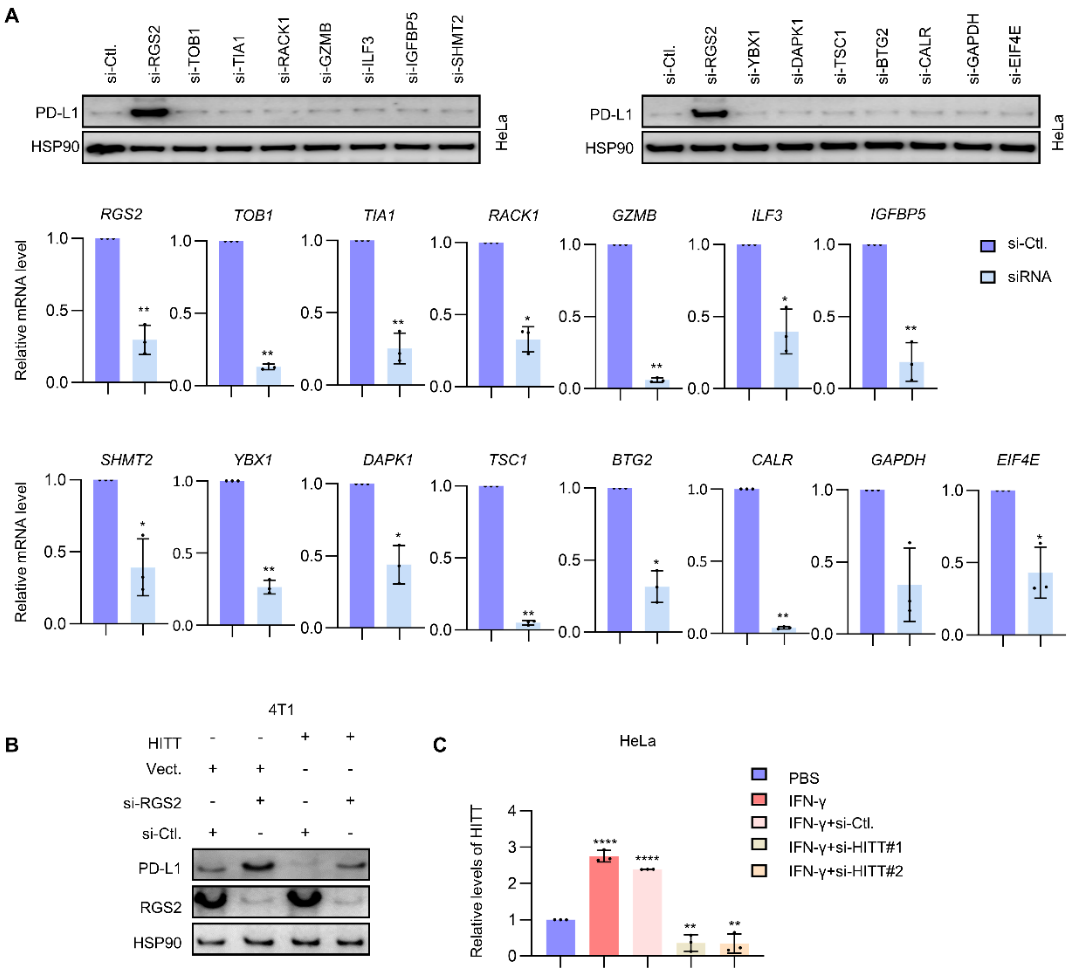

Supplemental Figure 4 HITT inhibits PD-L1 expression dependently on RGS2

(**A**) PD-L1 levels were measured by western blot following small interfering RNA (siRNA)-mediated genetic KD of the indicated targets (up). KD efficiencies were verified by qRT-PCR (bottom). (**B**) PD-L1 level was measured by western blot in 4T1 cells with or without RGS2 KD and/or HITT overexpression. (**C**) Expression rates of HITT were determined by qRT-PCR in HeLa cells used in Figure 4**C** and Figure 6**A** and representative data were present in **C**. Data are derived from three independent experiments shown as mean ± SEM. **P* < 0.05; ***P* < 0.01; *****P* < 0.0001 by Student's t test (**A**) and one-way ANOVA test (**C**).

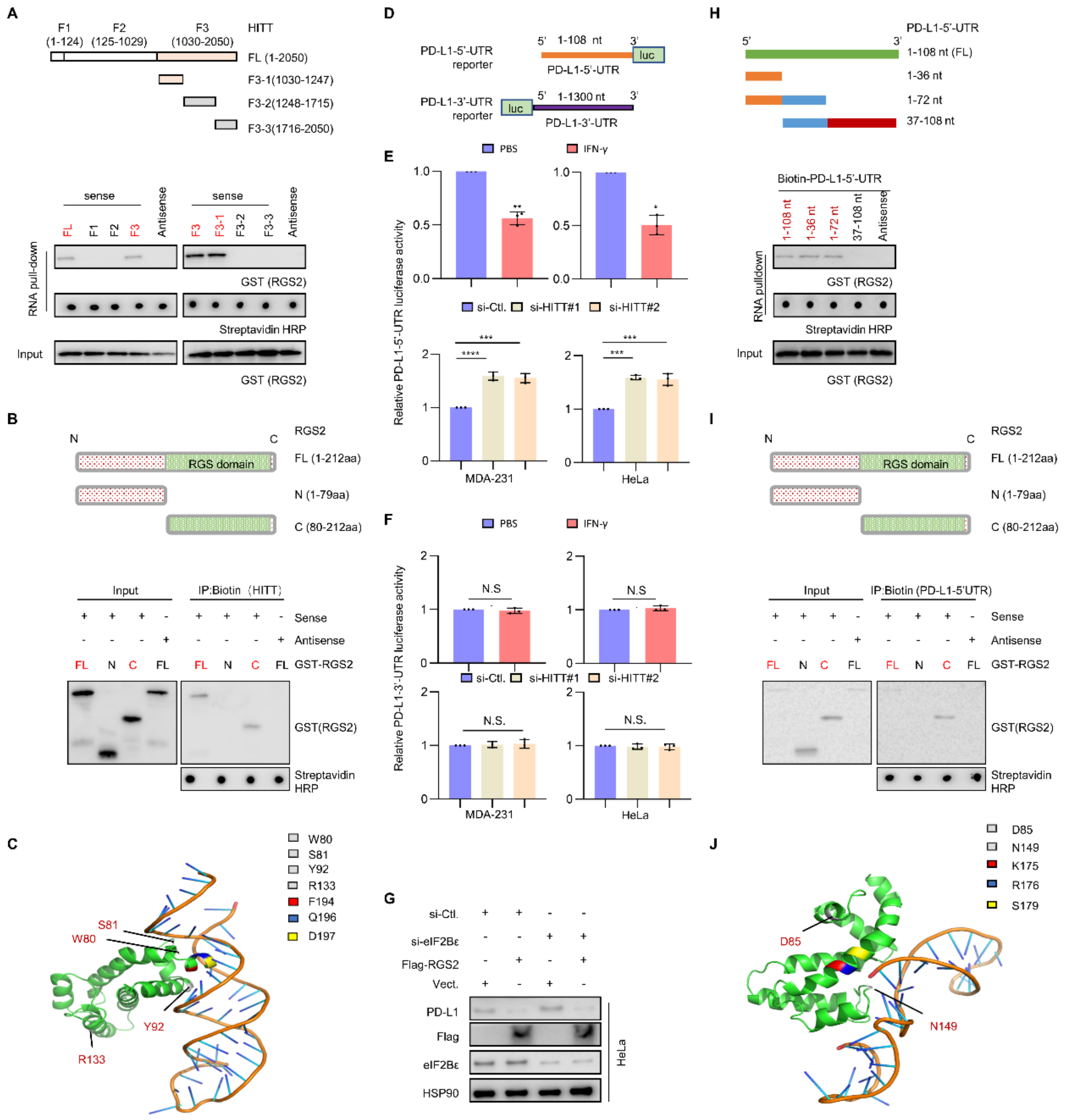

Supplemental Figure 5 The interaction mechanisms of HITT/*PD-L1* 5′-UTR/RGS2

(**A** and **B**) GST-tagged full length (FL) RGS2 protein (**A**) or its truncates (**B**) pulled down by Biotin-HITT (FL and HITT fragments) or Biotin-Antisense-HITT control were determined by western blot. Schematic showing HITT or RGS2 fragmented constructs in relation to FL HITT or RGS2 were shown in upper panels, respectively in **A** and **B**. (**C**) Representative model of RGS2 (80-212 aa)/HITT F3-1 (1080-1130 nt) complex predicted by HDOCK docking software. Seven most potential residues in mediating the binding with HITT F3-1 (1080-1130 nt) were highlighted in different colors. (**D**) Schematic of PD-L1-5′-UTR and PD-L1-3′-UTR luciferase reporter constructs. (**E** and **F**) PD-L1 -5′-UTR- (**E**) and PD-L1-3′-UTR- (**F**) controlled luciferase activities were determined by luciferase reporter assay in HITT overexpression stable lines or HITT KD cells. (**G**) PD-L1 levels were measured

by western blot under the indicated conditions. (**H** and **I**) GST-tagged-FL RGS2 protein (**H**) or its truncates (**I**) pulled down by Biotin-PD-L1-5′-UTR (FL and PD-L1-5′-UTR fragments) or Biotin-Antisense-PD-L1-5′-UTR control were determined by western blot. Schematic showing PD-L1-5′-UTR (**H**) or RGS2 fragmented (**I**) constructs in the upper panels, respectively. (**J**) Representative model of RGS2 (80-212 aa)/5′-UTR (1-36 nt) complex was predicted by HDOCK docking software. Five most potential residues in mediating the binding with 5′-UTR (1-36 nt) were highlighted in different colors. Data are derived from three independent experiments shown as mean ± SEM. **P* < 0.05; ***P* < 0.01; ****P* < 0.001; *****P* < 0.0001; N.S. not significant by Student's t test (**E**, **F**) and one-way ANOVA test (**E**, **F**).

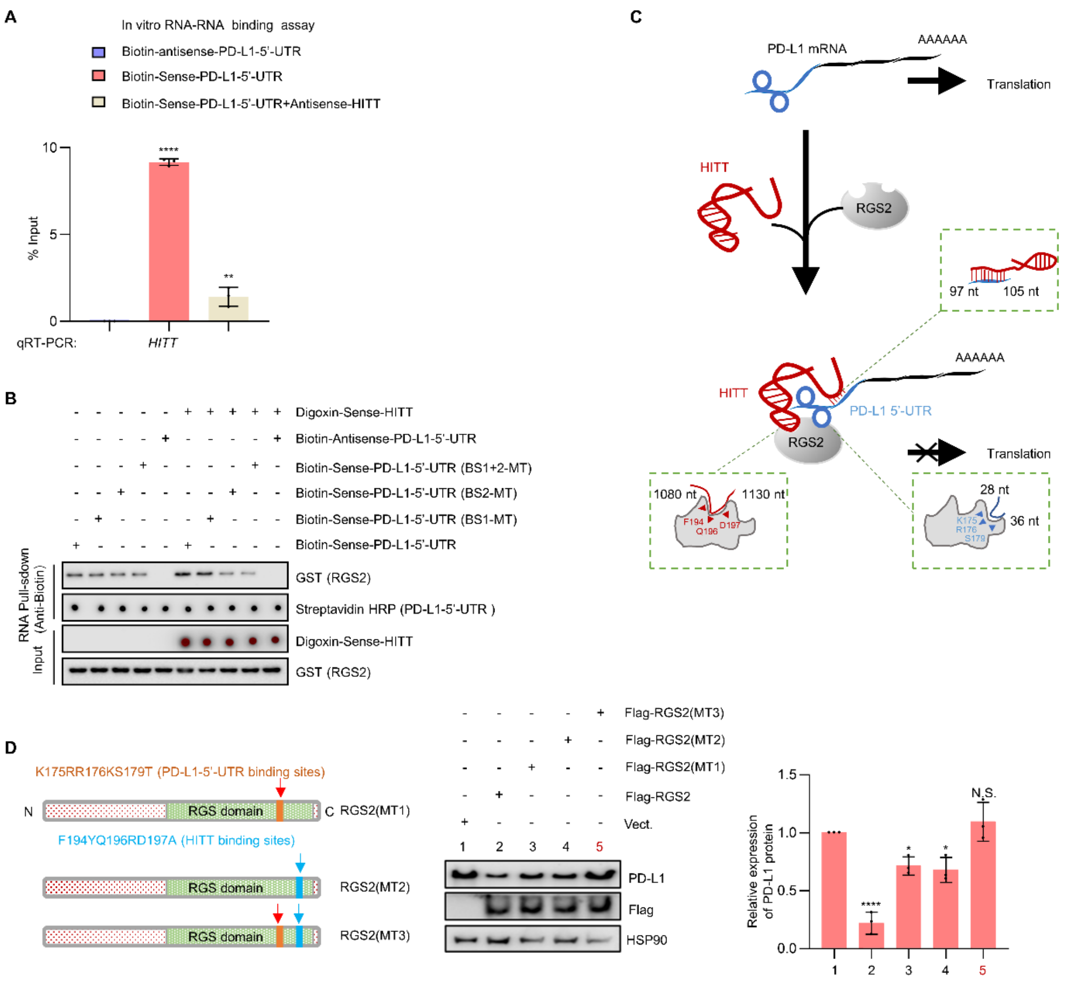

Supplemental Figure 6 HITT/*PD-L1* 5'-UTR/RGS2 interact with each other

(**A**) HITT co-precipitated by Biotin-PD-L1-5'-UTR or Biotin-Antisense-PD-L1-5'-UTR control was determined by qRT-PCR with or without Antisense-HITT. (**B**) GST-labeled full length (FL) RGS2 protein pulled down by Biotin-PD-L1-5'-UTR, -PD-L1-5'-UTR (BS1-MT), -PD-L1-5'-UTR (BS2-MT), -PD-L1-5'-UTR (BS1+2-MT) or Biotin-Antisense-PD-L1-5'-UTR control were determined by RNA pull down assay. (**C**) A schematic of molecular mechanisms underlying RGS2/HITT-inhibited PD-L1 translation. HITT (1080-1130 nt) and PD-L1-5'-UTR (28-36 nt) are essential for their binding with RGS2 at F194, Q196, D197 and K175, R176, S179, respectively. HITT (1030-1247 nt) also forms RNA-RNA duplex with PD-L1-5'-UTR (BS2-MT, 97-105 nt). These interactions are essential to attenuate the translation rate of PD-L1. (**D**) PD-L1 protein levels were analyzed by western blot assay in HeLa cells transfected with flag tagged RGS2 WT or its mutants (RGS2 MT1: K175RR176KS179T; RGS2 MT2: F194YQ196RD197A and RGS2 MT3: K175RR176KS179TF194YQ196RD197A). The PD-L1

intensities were quantified and shown in bar graph. Data are derived from three independent experiments shown as mean ± SEM. *P < 0.05; **P < 0.01; ***P < 0.001; ****P < 0.0001; N.S. not significant by one-way ANOVA test (**A**, **D**).

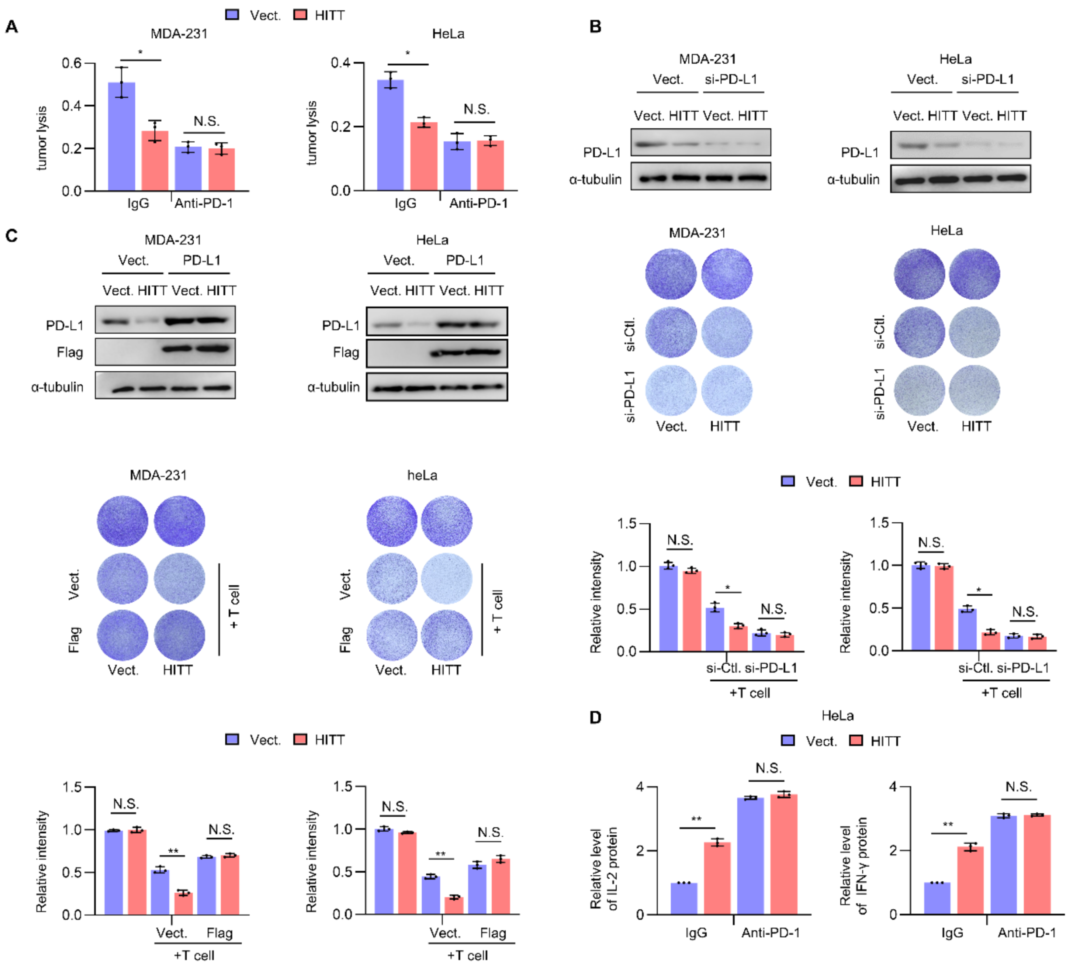

Supplemental Figure 7 PD-L1 reduction is required for the increased anti-tumour immunity mediated by HITT

(**A**) The viabilities of MDA-231 and Hela cells were determined by MTS assay following co-culture cancer cells with the activated T cell for 6h in the presence of IgG control or anti-PD-1 antibody after the HITT overexpression. (**B, C**) The attached survival MDA-231 or HeLa cells were detected by crystal violet staining following co-culture cancer cells with the activated T cell for 6h after the HITT overexpression with or without PD-L1 KD (**B**) or overexpression (**C**). The intensities were quantified and the relative levels were shown in bar graph (bottom). PD-L1 KD and overexpression efficiencies were determined by western blot (upper). (**D**) Detection of IL-2 and IFN-γ levels in the supernatants from the co-cultures of T-cell and cancer cell in the presence of absence of IgG control or anti-PD-1 antibody by ELISA assays. Data are derived from three independent

experiments shown as mean ± SEM. *$P < 0.05$; **$P < 0.01$; N.S. not significant by Student's t test (**A**-**D**).

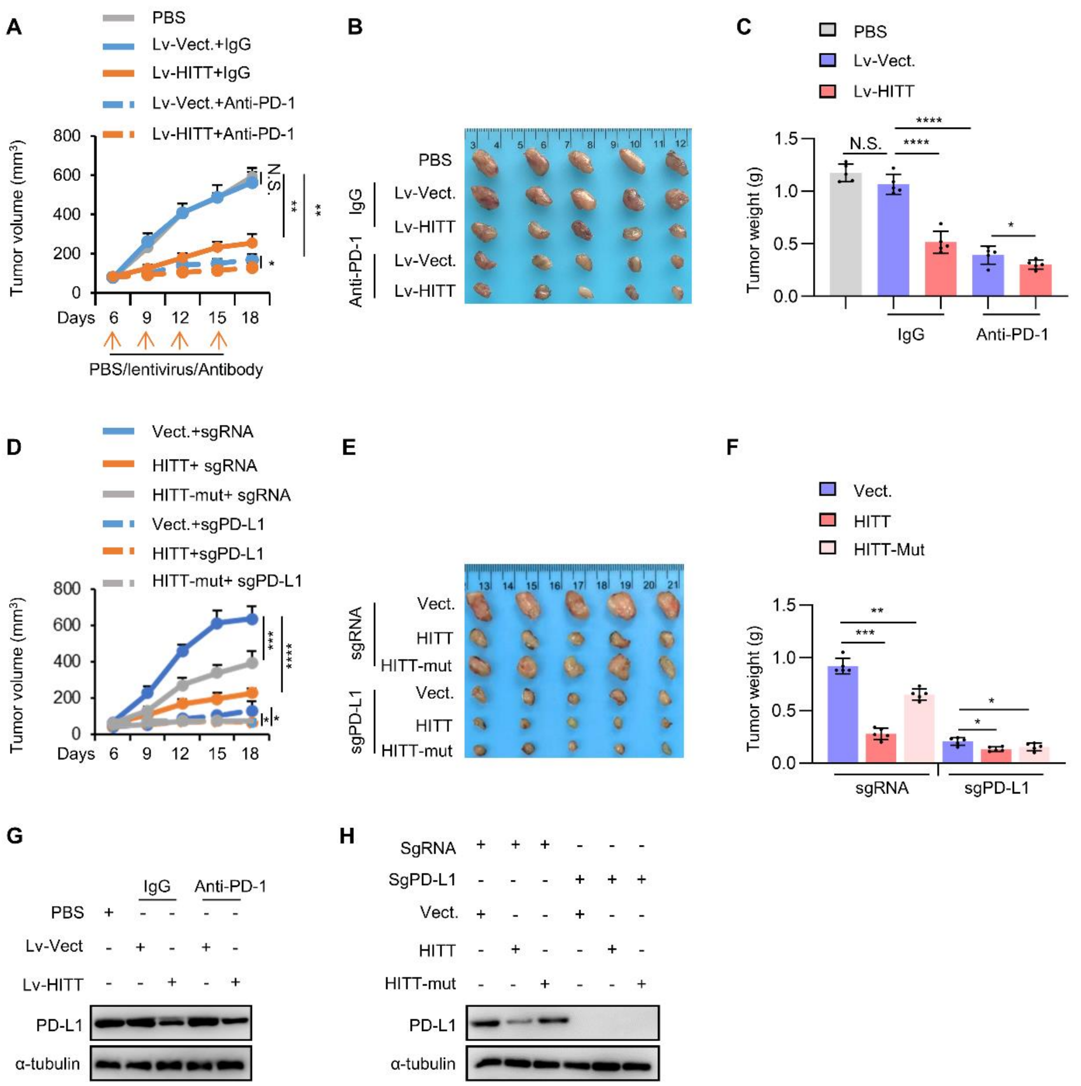

Supplemental Figure 8 HITT sensitizes cancer cells to T cell-mediated cytotoxicity through inhibiting PD-L1 expression

(**A-C**) 4T1 syngeneic tumors in immune-competent BALB/c mice were monitored following the administration of IgG control or anti-CD8α antibody and introducing PBS, control and HITT expressing lenti-virus. The tumour volume (**A**) and images of syngeneic tumor (**B**), and the tumor weight (**C**) were shown respectively. Each dot represents an evaluation in an individual tumor. (**D-F**) The tumour volume (**D**) and images of syngeneic tumor (**E**), and the tumor weight (**F**) of the 4T1/Vect, 4T1/HITT, 4T1/HITT-Mut, 4T1/Vect/PD-L1 KO, 4T1/HITT/PD-L1 KO, 4T1/HITT-Mut/PD-L1 KO syngeneic tumors in immune-competent BALB/c mice were shown respectively. Each dot represents an evaluation in an individual tumor. (**G**) PD-L1 protein of tumor samples from PBS,

lenti-Vect+IgG, lenti-HITT+IgG, Lenti-Vect+anti-PD-1, lenti-HITT+anti-PD-1 groups were determined by western blot. (**H**) PD-L1 protein levels were measured in 4T1/Vect, 4T1/HITT, 4T1/HITT-Mut, 4T1/Vect/PD-L1 KO, 4T1/HITT/PD-L1 KO, 4T1/HITT-Mut/PD-L1 KO six groups. HITT-Mut indicated the PD-L1 mRNA binding defective HITT. Data in **A**, **C**, **D** and **F** are shown as mean ± SD. **P* < 0.05; ***P* < 0.01; ****P* < 0.001; ****P* < 0.0001; N.S. not significant by two-way ANOVA test (**A**, **D**, n=5 mice per group) and one-way ANOVA test (**C**, **F** n=5 mice per group).

Supplemental Table 1. Gene background of cancer cell lines treated with IFN-γ.

Cell line	TNBC	Metastatic
MDA-MB-231	YES	Pleural effusion
MDA-MB-453	NO	Pericardial effusion
MDA-MB-468	YES	Pleural effusion
BT549	YES	Pleural effusion
MCF7	NO	Pleural effusion
T47D	NO	Pleural effusion

Supplemental Table 2. Proteins have been annoated to negatively regulate protein translation in Gene Ontology database.

UnprotKB Accession	Gene	UnprotKB	Gene
UniProtKB:P41220	RGS2[1]	UniProtKB:Q9BWF3	RBM4
UniProtKB:P50616	TOB1[2]	UniProtKB:Q6IMN6	CAPRIN
UniProtKB:P31483	TIA1[3]	UniProtKB:Q9BZC1	CELF4
UniProtKB:P63244	RACK1[4]	UniProtKB:C9JE40	PATL2
UniProtKB:P10144	GZMB[5]	UniProtKB:P60323	NANOS
UniProtKB:Q12906	ILF3[6]	UniProtKB:P60321	NANOS
UniProtKB:P24593	IGFBP5[7]	UniProtKB:P51114	FXR1
UniProtKB:P34897	SHMT2[8]	UniProtKB:Q9NZI8	IGF2BP
UniProtKB:P67809	YBX1[9]	UniProtKB:Q8ND56	LSM14
UniProtKB:P53355	DAPK1[10]	UniProtKB:Q2Q1W2	TRIM71
UniProtKB:Q92574	TSC1[11]	UniProtKB:Q8WU17	RNF139
UniProtKB:P78543	BTG2[12]	UniProtKB:P19338	NCL
UniProtKB:P27797	CALR[13]	UniProtKB:Q9H2U1	DHX36
UniProtKB:P04406	GAPDH[14]	UniProtKB:O43293	DAPK3
UniProtKB:P06730	EIF4E[15]	UniProtKB:O00571	DDX3X
UniProtKB:Q9NRA8	EIF4ENIF	UniProtKB:P48200	IREB2
UniProtKB:O60573	EIF4E2	UniProtKB:P00374	DHFR
UniProtKB:Q9NZJ5	EIF2AK3	UniProtKB:P40429	RPL13A
UniProtKB:Q96EH3	MALSU1	UniProtKB:P26196	DDX6
UniProtKB:P19525	EIF2AK2	UniProtKB:Q14451	GRB7
UniProtKB:Q9ULR5	PAIP2B	UniProtKB:Q9H9A5	CNOT1
UniProtKB:Q9BPZ3	PAIP2	UniProtKB:O60506	SYNCRI
UniProtKB:Q8NDQ6	ZNF540	UniProtKB:Q9Y2Y8	PRG3
UniProtKB:Q8NE35	CPEB3	UniProtKB:Q9NRR6	INPP5E
UniProtKB:P51116	FXR2	UniProtKB:Q00577	PURA
UniProtKB:Q14011	CIRBP	UniProtKB:O14682	ENC1
UniProtKB:O00425	IGF2BP3	UniProtKB:Q92600	CNOT9
UniProtKB:Q5PRF9	SAMD4B	UniProtKB:Q9UFF9	CNOT8
UniProtKB:Q06787	FMR1	UniProtKB:Q6Y7W6	GIGYF2
UniProtKB:Q9UPU9	SAMD4A	UniProtKB:A5YKK6	CNOT1
UniProtKB:Q9Y6M1	IGF2BP2	UniProtKB:P03950	ANG
UniProtKB:Q9H9Z2	LIN28A	UniProtKB:P04818	TYMS
UniProtKB:Q14444	CAPRIN1	UniProtKB:Q9UIV1	CNOT7
UniProtKB:Q9H4Z3	PCIF1	UniProtKB:P38919	EIF4A3
UniProtKB:Q9BYD1	MRPL13	UniProtKB:P23396	RPS3
UniProtKB:Q8WY41	NANOS1	UniProtKB:Q8IZH2	XRN1
UniProtKB:Q96PZ0	PUS7	UniProtKB:P52758	RIDA
UniProtKB:Q6PKG0	LARP1	UniProtKB:P34896	SHMT1
UniProtKB:P10276	RARA	UniProtKB:P07814	EPRS1

Green: proteins that have the potential in immune regulation

Supplemental Table 3. The prediction of the RNA binding sites in RGS2 by HDOCK online software

Potential PD-L1-5′-UTR binding sites	Frequency predicted by the top 10 models	Potential HTT binding sites	Frequency predicted by the top 10 models
N175	9	D197	7
R176	9	Q196	6
D85	6	F194	6
S179	5	S81	6
N149	5	W80	5
E191	4	Y92	5
R188	4	R133	5
N183	4	C199	4
E182	4	E193	4
Q153	4	G168	4
F152	4	Q160	4
E104	4	Q126	4
E86	4	P125	4
W80	4	K123	4
		S103	4
		A99	4
		E82	4

Only those residues predicated by more than 4 times in the top 10 models were listed in the table

Yellow: The residues predicted by more than 5 times

Supplemental Table 4. Plasmids used in this study

No.	Plasmid	Source
1	pCDNA3.1-HA-E2F1	This study
2	pLncKP--HITTT	This study
3	pGL3-HITT-Promoter-luc (promoter luciferase reporter)	This study
4	pGL3-HITT-Promoter-MT1-luc	This study
5	pGL3-HITT-Promoter-MT2-luc	This study
6	pMIR-luc-HITT (Luciferase-HITT reporter)	This study
7	pCDNA3.1-FL (1-2050 nt)	This study
8	pCDNA3.1-F1 (1-214 nt)	This study
9	pCDNA3.1-F2 (125-1029 nt)	This study
10	pCDNA3.1-F3 (1030-2050 nt)	This study
11	pCDNA3.1-F3-1 (1030-1247 nt)	This study
12	pCDNA3.1-F3-1.1 (1030-1130nt)	This study
13	pCDNA3.1-F3-1.2 (1080-1180nt)	This study
14	pCDNA3.1-F3-1.3 (1130-1230nt)	This study
15	pCDNA3.1-F3-1.4 (1180-1247nt)	This study
16	pCDNA3.1-F3-2 (1248-1715nt)	This study
17	pCDNA3.1-F3-3 (1716-2050nt)	This study
18	pCDNA3.1-HITT-del(1080-1130nt)	This study
19	pGL3-3'UTR reporter WT 1.3 kb CD274	Addgene#107009
20	PGL3-PD-L1-5’-UTR-luc	This study
21	PGL3-PD-L1-5’-UTR-(1-36 nt)-luc	This study
22	PGL3-PD-L1-5’-UTR-(37-108 nt)-luc	This study
23	PGL3-PD-L1-5’-UTR-(1-36 nt)-MT1-luc	This study
24	PGL3-PD-L1-5’-UTR-(1-36 nt)-MT2-luc	This study
25	PGL3-PD-L1-5’-UTR-(1-36 nt)-MT3-luc	This study

continued

No.	Plasmid name	Source
26	PGL3-PD-L1-5′-UTR-(1-36 nt)-MT4-luc	This study
27	PGL3-PD-L1-5′-UTR-BS1-MT-luc	This study
28	PGL3-PD-L1-5′-UTR-BS2-MT-luc	This study
29	PGL3-PD-L1-5′-UTR-BS1+2-MT-luc	This study
30	pCDNA3.1-PD-L1-5′-UTR-(1-108 nt)	This study
31	pCDNA3.1-PD-L1-5′-UTR-(1-36 nt)	This study
32	pCDNA3.1-PD-L1-5′-UTR-(1-72 nt)	This study
33	pCDNA3.1-PD-L1-5′-UTR-(37-108 nt)	This study
34	pCDNA3.1-PD-L1-5′-UTR-MT1	This study
35	pCDNA3.1-PD-L1-5′-UTR-MT2	This study
36	pCDNA3.1-PD-L1-5′-UTR-MT3	This study
37	pCDNA3.1-PD-L1-5′-UTR-MT4	This study
38	pCDNA3.1-PD-L1-5′-UTR-BS1-MT	This study
39	pCDNA3.1-PD-L1-5′-UTR-BS2-MT	This study
40	pCDNA3.1-PD-L1-5′-UTR-BS1+2-MT	This study
41	pCDNA3.1-Flag-RGS2	This study
42	pCDNA3.1-Flag-RGS2-MT1 (K175RR176KS179T)	This study
43	pCDNA3.1-Flag-RGS2-MT2 (F194YQ196RD197A)	This study
44	pCDNA3.1-Flag-RGS2-MT3 (K175RR176KS179T-F194YQ196RD197A)	This study
45	pGEX6P-1-Flag-RGS2-FL(1-212 aa)	This study
46	pGEX6P-1-Flag-RGS2-N (1-79 aa)	This study
47	pGEX6P-1-Flag-RGS2-C (80-212 aa)	This study
48	pGEX6P-1-Flag-RGS2(W80F)	This study
49	pGEX6P-1-Flag-RGS2(S81T)	This study
50	pGEX6P-1-Flag-RGS2(Y92F)	This study

continued

No.	Plasmid	Source
51	pGEX6P-1-Flag-RGS2(R133K)	This study
52	pGEX6P-1-Flag-RGS2(F194Y)	This study
53	pGEX6P-1-Flag-RGS2(Q196R)	This study
54	pGEX6P-1-Flag-RGS2(D197A)	This study
55	pGEX6P-1-Flag-RGS2(W80FS81T)	This study
56	pGEX6P-1-Flag-RGS2(F194YQ196RD197A)	This study
57	pGEX6P-1-Flag-RGS2(D85A)	This study
58	pGEX6P-1-Flag-RGS2(N149I)	This study
59	pGEX6P-1-Flag-RGS2(K175R)	This study
60	pGEX6P-1-Flag-RGS2(R176K)	This study
61	pGEX6P-1-Flag-RGS2(S179T)	This study
62	pGEX6P-1-Flag-RGS2(K175RR176KS179T)	This study

Supplemental Table 5. Primers sequences used for qRT-PCR in this study

Name	Forword sequence (5'-3')	Reverse sequence (5'-3')
HITT	ACACAAATGCTGGCCTCTGTCA	GGCAAGTGGCAAAGCCTCTC
PD-L1	ATGGTGGTGCCGACTACAAG	GGAATTGGTGGTGGTGGTCT
PD-L1-5'UTR	GGCGCAACGCTGAGCAGC	CTTTCTGGAATGCCCT
HITT-F1	GGTCCCTGTCCTCACAGAGTT	TGTTCTTGTCTATCGTCTTCTTGC
HITT-F2	AAGGAGGGTAGGAGTCTTGGTC	CCGAAATAAAGGCAGGAGTGA
HITT-F3	CCAAAAGGCAAAAGCAGGGTG	CAGGAAGGCTCTGGCTCAGGAAT
HITT-F3-1	CTCCCTCTCCTGCCTTTGACCTC	CTCGTCCTCCCAGATTGATGCTC
HITT-F3-2	GTAGGGGGTTTCTGTAGTATGAGAC	GTATTTTGGGGTTCTGTTAAAATG
HITT-F3-3	TCTTAGAGAGACCCCTCACCCCTTC	CGTCCGGCTAATTTTTGTGTTTTTA
RGS2	CTCTACTCCTGGGAAGCCCAAA	TTGCTGGCTAGCAGCTCGTCAA
TOB1	CTCCATCGCCTCCTTTTGGT	CCTTGTTGCTACGGCCACTA
TIA1	GGACGAGATGCCCAAGACTC	GGGGTTGTTGCCCAATTCAC
RACK1	GGGGTCACTCCCACTTTGTT	AATCTGCCGGTTGTCAGAGG
GZMB	GATCATCGGGGGACATGAGG	ATGGAGCTTCCCCAACAGTG
ILF3	AGCCACATAACCCTGGCTTC	ACAAAACCTGTGTAGCCTGC
IGFBP5	CCCAATTGTGACCGCAAAGG	CGTCAACGTACTCCATGCCT
SHMT2	CTCTTTGTTTTGGGCGGCTC	GACACTGCCTGTCCTTCTCC
YBX1	GGGCTTATCCCGCCTGTC	ACCTTCGTTGCGATGACCTT
DAPK1	CAGTGGACAGTTTGCGGTTG	GATGACATTGGGGTGCTGGA
TSC1	CTGACACACCAAAGCAAGCC	TCCATTGGGGAGGTAGAGGG
BTG2	GAGGTGTCCTACCGCATTGG	CACGTAGTTCTTGGAGGGGC
CALR	GTACACACTGATTGTGCGGC	TTCTTGGGTGGCAGGAAGTC
EIF4E	GTAGCGCACACTTTCTGGA	CAGAGTGCCCATCTGTTCTGT
18S	AACTTTCGATGGTAGTCGCCG	CCTTGGATGTGGTAGCCGTTT

Supplemental Table 6. Reagents used in this study

Reagent	Manufacturer	No
PD-L1	Cell Signaling Technology	13684S
PD-L2	Cell Signaling Technology	82723S
RGS2	Santa Cruz	SC-100761
E2F1	Cell Signaling Technology	3742S
EGR1	Santa Cruz	SC-189
TAF-1	Proteintech	20260-1-AP
Biotin	abcam	ab53494
α-tubulin	Proteintech	66031-1-Ig
β-actin	Proteintech	60008-1-Ig
GAPDH	Proteintech	60004-1-Ig
HSP70	Proteintech	10995-1-AP
HSP90	Proteintech	13171-1-AP
P62	Proteintech	18420-1-AP
FLAG	Bio Rad	MCA4764
GST	Abclonal	AE006
Goat anti-Rabbit IgG	Thermo Fisher Scientific	31460
Goat anti-Mouse IgG	Thermo Fisher Scientific	31430
FITC anti-human PD-L1	Biolegend	393605
PE-anti-Mouse CD3ε	Biolegend	100307
PE-Cyanine 7 anti-Mouse IFN-γ	Biolegend	505825
Rat anti-mouse CD8α	BD Pharmingen TM	568164
Alexa Fluor 488 anti-human IgG	Biolegend	M1310G05
anti-human IgG (H+L) Alexa Fluor 488	Thermo Fisher Scientific	A-11013
anti-mouse CD8α antibody	Bio X Cell	BE0117
anti-mouse PD-1	Bio X Cell	BP0146
anti-human-CD3	Biolegend	317326
anti-human-CD28	Biolegend	302934
Human IFN-γ	R&D Systems	34992
Human IL-2	R&D Systems	202-IL
Rat IL-2	Miltenyi	130-127-428
OVA257-264 peptide	Sigma	S7951
Duolink In Situ PLA Probe Anti-Rabbit	Sigma	Duo92002
Duolink In Situ PLA Probe Anti-Mouse	Sigma	Duo92004
Duolink In Situ Detection Reagents Red	Sigma	Duo92008
mouse CD8+ MicroBeads	Miltenyi	130-116-478

Supplemental Table 7. Probe sequences used in this study

Probe name	Sequence (5'-3')
HITT-PLA	AGGTGGAGGCCATAGACTTGCTGTGTGCAGGGACCAAGACTTAGGGTGGG
HITT del(1080-1130 nt)-PLA	CCGCATCGCTCCCTCTCCTGCCTTTGACCTCCCACAGGGCACCTCCACAT
HITT-FISH	AGGTGGAGGCCATAGACTTGCTGTGTGCAGGGACCAAGACTTAGGGTGGG
PD-L1-5'-UTR-PLA	GAGAGCTGGTCCTTCAACAGCTGATCATGCAGCGGTACACCTCAGTGTGC
PD-L1-5'-UTR(28-36 nt-MT)-PLA	CCGCGTTGCGACTCGTCGACCGCGCAGCCGCGCGGC
PD-L1-5'-UTR-FISH	GAGAGCTGGTCCTTCAACAGCTGATCATGCAGCGGTACACCTCAGTGTGC

References

1. Oliveira-Dos-Santos AJ, Matsumoto G, Snow BE, Bai D, Houston FP, Whishaw IQ, et al. Regulation of T cell activation, anxiety, and male aggression by RGS2. *Proc Natl Acad Sci U S A.* 2000;97(22):12272-7.
2. Hosokawa K, Kajigaya S, Keyvanfar K, Qiao W, Xie Y, Townsley DM, et al. T Cell Transcriptomes from Paroxysmal Nocturnal Hemoglobinuria Patients Reveal Novel Signaling Pathways. *J Immunol.* 2017;199(2):477-88.
3. Meyer C, Garzia A, Mazzola M, Gerstberger S, Molina H, and Tuschl T. The TIA1 RNA-Binding Protein Family Regulates EIF2AK2-Mediated Stress Response and Cell Cycle Progression. *Mol Cell.* 2018;69(4):622-35.e6.
4. Chou WC, Guo Z, Guo H, Chen L, Zhang G, Liang K, et al. AIM2 in regulatory T cells restrains autoimmune diseases. *Nature.* 2021;591(7849):300-5.
5. Golstein P, and Griffiths GM. An early history of T cell-mediated cytotoxicity. *Nat Rev Immunol.* 2018;18(8):527-35.
6. Alriyami M, Marchand L, Li Q, Du X, Olivier M, and Polychronakos C. Clonal copy-number mosaicism in autoreactive T lymphocytes in diabetic NOD mice. *Genome Res.* 2019;29(12):1951-61.
7. Egbeto IA, Garelli CJ, Piedra-Mora C, Wong NB, David CN, Robinson NA, et al. Case Series: Gene Expression Analysis in Canine Vogt-Koyanagi-Harada/Uveodermatologic Syndrome and Vitiligo Reveals Conserved Immunopathogenesis Pathways Between Dog and Human Autoimmune Pigmentary Disorders. *Front Immunol.* 2020;11:590558.
8. Ron-Harel N, Santos D, Ghergurovich JM, Sage PT, Reddy A, Lovitch SB, et al. Mitochondrial Biogenesis and Proteome Remodeling Promote One-Carbon Metabolism for T Cell Activation. *Cell Metab.* 2016;24(1):104-17.
9. Zhao P, Ji MM, Fang Y, Li X, Yi HM, Yan ZX, et al. A novel lncRNA TCLlnc1 promotes peripheral T cell lymphoma progression through acting as a modular scaffold of HNRNPD and YBX1 complexes. *Cell Death Dis.* 2021;12(4):321.
10. Chou TF, Chuang YT, Hsieh WC, Chang PY, Liu HY, Mo ST, et al. Tumour suppressor death-associated protein kinase targets cytoplasmic HIF-1α for Th17 suppression. *Nat Commun.* 2016;7:11904.
11. Shi L, Chen X, Zang A, Li T, Hu Y, Ma S, et al. TSC1/mTOR-controlled metabolic-epigenetic cross talk underpins DC control of CD8+ T-cell homeostasis. *PLoS Biol.* 2019;17(8):e3000420.
12. Hwang SS, Lim J, Yu Z, Kong P, Sefik E, Xu H, et al. mRNA destabilization by BTG1 and BTG2 maintains T cell quiescence. *Science.* 2020;367(6483):1255-60.
13. Blees A, Januliene D, Hofmann T, Koller N, Schmidt C, Trowitzsch S, et al. Structure of the human MHC-I peptide-loading complex. *Nature.* 2017;551(7681):525-8.
14. Mondragón L, Mhaidly R, De Donatis GM, Tosolini M, Dao P, Martin AR, et al. GAPDH Overexpression in the T Cell Lineage Promotes Angioimmunoblastic T Cell Lymphoma through an NF-κB-Dependent Mechanism. *Cancer Cell.* 2019;36(3):268-87.e10.
15. Yi W, Gupta S, Ricker E, Manni M, Jessberger R, Chinenov Y, et al. The mTORC1-4E-BP-eIF4E

axis controls de novo Bcl6 protein synthesis in T cells and systemic autoimmunity. *Nat Commun.* 2017;8(1):254.
